# Gαi1/3 signaling mediates IL-5-induced eosinophil activation and type 2 inflammation in eosinophilic chronic rhinosinusitis

**DOI:** 10.3389/fimmu.2024.1460104

**Published:** 2025-01-07

**Authors:** Huanxia Xie, Jiang Ji, Zhichen Liu, Ning Lu, Yuqian Wei, Aina Zhou, Jisheng Liu, Qingqing Jiao

**Affiliations:** ^1^ Department of Ear, Nose, and Throat, The First Affiliated Hospital of Soochow University, Suzhou, China; ^2^ Department of Dermatology, The Second Affiliated Hospital of Soochow University, Suzhou, China; ^3^ Central Research Laboratory, The First Affiliated Hospital of Soochow University, Suzhou, China; ^4^ Department of Dermatology, The First Affiliated Hospital of Soochow University, Suzhou, China

**Keywords:** Gαi1, Gαi3, chronic rhinosinusitis, eosinophilic chronic rhinosinusitis, eosinophils, type 2 inflammation, IL-5

## Abstract

**Background:**

Uncontrolled severe eosinophilic chronic rhinosinusitis (eCRS) is associated with elevated levels of Th2 cells and raised immunoglobulin concentrations in nasal polyp tissue. eCRS is characterized by high eosinophilic infiltration and type 2 inflammation. Gαi1/3 proteins participate in allergic inflammation by regulating immune cells. Whether Gαi1/3 proteins have a role in the development of eCRS remains unknown.

**Objectives:**

To investigate the association between Gαi1/3 expression levels and CRS and the underlying mechanisms

**Methods:**

Western blotting and immunohistology were used to detect Gαi1/3 expression. Correlations between Gαi1/3 and immune cells and clinical parameters were analyzed. Signaling pathway activation in IL-5-induced Gαi1/3-knockout or knockdown mouse embryonic fibroblasts (MEFs) and eosinophils (EoL-1 cells) was detected by western blotting. EdU/DAPI was used to evaluate the proliferation of EoL-1 cells. A CRS model was established using Gαi1/3-knockout mice, and histological analysis and inflammatory cytokine measurements were performed.

**Results:**

Compared with the non-eCRS subset, the eCRS subset showed significantly increased Gαi1/3 expression levels. High nasal tissue Gαi1/3 levels were linked to high tissue eosinophil infiltration, and high disease severity and allergic conditions in CRS patients. Gαi1/3 were required for IL-5-induced Akt-mTOR and Erk activation in MEFs. In EoL-1 cells, Gαi1/3 was associated with IL-5-activated IL-5Rα, promoting IL-5Rα endocytosis and transducing downstream signaling. IL-5-induced EoL-1 cell proliferation and degranulation were suppressed after Gαi1/3 silencing. In a CRS murine model, immune cell infiltration and type 2 inflammation were largely impaired in Gαi1/3-double-knockout mice.

**Conclusion:**

Increased Gαi1/3 expression levels in nasal tissue are linked to eosinophil infiltration and increased disease severity in CRS patients. Gαi1/3 contributes to eosinophil activation and participates in regulating allergic inflammation in CRS patients.

## Introduction

Chronic rhinosinusitis (CRS) is defined as local inflammation of the nasal and paranasal sinus mucosae with symptoms including nasal congestion, loss of smell, rhinorrhea, and facial pain that persist for at least 12 consecutive weeks, leading to a significant burden on society in terms of healthcare costs and productivity loss (1, 2). CRS is classified as chronic rhinosinusitis with nasal polyps (CRSwNP) or without nasal polyps (CRSsNP). Based on the extent of tissue eosinophilia, the clinical phenotypes are predominantly eosinophilic chronic rhinosinusitis (eCRS) and non-eosinophilic chronic rhinosinusitis (non-eCRS), as determined by histological eosinophil quantification. Compared with non-eCRS, eCRS is associated with more frequent comorbid asthma, severe clinical symptoms, and a higher ratio of recurrence and revision surgery (3). According to the European Position Paper on Rhinosinusitis and Nasal Polyps 2020 (EPOS2020) guidelines, eCRS is type 2 dominant and is defined histologically as an elevated tissue eosinophil count > 10 cells/high-power field (4). CRS research has revealed that patients with a pure or mixed type 2 endotype tend to be much more resistant to current therapies, exhibiting a high recurrence rate when compared with pure type 1 or 3 endotypes (4).

The immune response more involves Th2 cells, eosinophils, and IgE, among others in eCRS group, suggesting greater eosinophil stimulation and chemotaxis, with a higher degree of Th2 inflammation (5, 6). Severe eCRS is often complicated by aspirin intolerance (7). Eosinophils interact with, Staphylococcal enterotoxin B, and fungi, all of which were found in the tissue of CRS patients. These interactions activate Th2 immune responses in the sinonasal mucosa and exacerbate local inflammation (3). The proliferation, differentiation, and activation of some cells, such as macrophages, human umbilical vein endothelial cells, and neuronal cells, require Gαi1/3 (8–10). Recently, Gαi1/3 proteins have been shown to participate in regulating immune cell function and affecting the development of allergic inflammation (10, 11). Thus, it would be interesting to explore whether Gαi1/3 can regulate eosinophils and thus affect Th2 cells.

G-protein-coupled receptors (GPCRs) are a large family of membrane protein receptors that recognize and bind to chemical substances in the surrounding environment, activating signaling pathways that induce changes in the organism (12). G proteins consist of three subunits: Gα, Gβ, and Gγ, with Gα being the primary functional subunit. Gβ and Gγ form a tightly bound dimer that associates with Gα, anchoring the complex within the membrane. There is a wide variety of G proteins, which can be categorized into four main classes based on the Gα subunit: Gαs, Gαi, Gαq, and Gα12. Gαi proteins contain three primary subunits, Gαi1, Gαi2, and Gαi3. Among these, Gαi1 and Gαi3 are highly expressed in the immune system and play roles in signaling processes for various receptors (13). They associate with GPCRs and inhibit adenylyl cyclase (AC). Studies have shown that Gαi1/3 proteins are essential novel proteins in transducing signals by various receptor tyrosine kinases (RTKs) (8, 9, 14–16) and also some non-RTK receptors (10, 17). In certain Th2 inflammatory diseases, Gαi1/3 primarily participates in immune regulation rather than Gαi2. Wei et al. (11) previously demonstrated that Gαi1/3 participates in regulating the degranulation of mast cells (MCs) and affects the occurrence and development of inflammation. Gαi1/3 knockdown in bone marrow-derived macrophage cells (BMDMs) significantly suppressed TNF-α, IL-6, and IL-12 production in response to lipopolysaccharide (18). Moreover, following IL-4 stimulation, Gαi1/3 associated with the intracellular domain of IL-4Rα promotes IL-4Rα endosomal trafficking and Gab1-Akt activation in BMDMs, to mediate type 2 immunity, inflammation, and allergy (10). These immune cells play important roles in the progression of CRS (19). It is, therefore, reasonable to speculate that Gαi protein may be involved in the regulation of type 2 inflammation, immunity, and allergy in eCRS; however, there is little direct evidence for the relationship between CRS and Gαi1/3.

Therefore, in the current study, we investigated the association between Gαi1/3 expression levels and CRS, especially eCRS. Correlations between Gαi1/3 levels and immune cell counts and clinical parameters were analyzed. We explored the activation of signaling pathways and cell proliferation and degranulation in a granulocyte macrophage colony-stimulating factor (GM-CSF)- and IL-5-induced Gαi1/3-knockdown eosinophil cell line (EoL-1 cells). An eosinophilic CRSwNP murine model was established using Gαi1/3-knockout mice, and histological analysis was performed, followed by the measurement of type 2 inflammatory cytokine levels.

## Methods

### Materials and reagents

*Staphylococcus aureus* enterotoxin B (SEB, S4881) and ovalbumin (OVA; S7951) were purchased from Sigma-Aldrich (St. Louis, MO, USA). Mouse IL-4 (EMC003.96), IL-5 (EMC108.96), IL-13 (EMC124.96), and IgE (EMC117.96) were purchased from NeoBioscience (Shenzhen, China). The mouse CCL-11 enzyme-linked immunosorbent assay (ELISA) kit (EK2130) was purchased from MultiSciences (Hangzhou, China). The mouse OVA-specific IgE ELISA kit (439807) was purchased from BioLegend (San Diego, CA, USA). Fetal bovine serum (FBS; 16140071) and other reagents for cell culture were purchased from Gibco BRL (Grand Island, NY, USA). Antibodies against Gαi1 (sc-13533), Gαi2 (sc-13534), Gαi3 (sc-365422), Gab1 (sc-133191), Akt (sc-81434), Erk1/2 (sc-514302), S6K (sc-8418), p-Gab1 (AP0256), p-Akt s473 (sc-101629), p-Erk1/2 (sc-136521), p-S6K (sc-8416), STAT5 (sc-74442), p-STAT5 (AP-0887), IL-5Rα (bs-2601R-100ul), IgE (ab75673), and tryptase (ab2378) were purchased from Bioss (Woburn, MA, USA), ABcolnal (Wuhan, China), Abcam (Cambridge, MA, USA), and Santa Cruz Biotechnology (Santa Cruz, CA, USA). IL-5 (CI59) and GM-CSF (CC79) were purchased from Novoprotein (Suzhou, China). Primers for polymerase chain reaction (PCR) were purchased from Sangon Biotech (Shanghai, China) and their sequences are listed in **Table 1**.

**Table 1:** Primers used in the study.

Molecule	Forward	Reverse
**ECP**	CCCACAGTTTACGAGGGCTC	ACCCGGAATCTACTCCGATGA
**PU.1**	GCGACCATTACTGGGACTTCC	GGGTATCGAGGACGTGCAT
**IL-4**	GGTCTCAACCCCCAGCTAGT	GCCGATGATCTCTCTCAAGTGAT
**IL-5**	GGGCTTCCTGCTCCTATCTA	CAGTCATGGCACAGTCTGAT
**IL-13**	CCTGGCTCTTGCTTGCCTT	GGTCTTGTGTGATGTTGCTCA
**CCL11/eotaxin**	CTGCTTGATTCCTTCTCTTTCCTAA	GGAACTACATGAAGCCAAGTCCTT
**Gαi1**	TTAGGGCTATGGGGAGGTTGA	GGTACTCTCGGGATCTGTTGAAA
**Gαi3**	GACGGCTAAAGATTGACTTTGGG	CCGTTTAATCACTCCTGCTAGTT

ECP, eosinophil cationic protein; PU.1, spi-1 proto-oncogene; IL-4, interleukin-4; IL-5, interleukin-5; IL-13, interleukin-13; CCL11, eotaxin-1; Gαi, Gα inhibitory subunit.

### Study subjects

Written informed consent was obtained from all patients. This study was approved by the Ethics Committee of the First Affiliated Hospital of Soochow University (approval no. 056). A total of 78 patients with CRS and 10 control subjects were enrolled in this study. The clinical characteristics of the patients are shown in **Table 2**. The diagnosis of sinus disease was based on clinical symptoms and related examinations according to the EPOS2020 guidelines (4). Patients without a history of CRS who underwent nasal surgery for various non-CRS indications were enrolled as controls. A positive skin prick test result indicated atopy. Diseased sinus mucosal samples from CRSsNP patients, polyp tissues from CRSwNP patients, and uncinate process tissues from control subjects were obtained during surgery. After the excised tissue is cleaned, it should be immediately fixed in 10% neutral buffered formalin and stored at room temperature for transport to the laboratory. Rhinology specialists classified CRS into CRSwNP and CRSsNP through nasal endoscopy and computed tomography (CT). CRSwNP was defined as eosinophilic when an elevated tissue eosinophil count > 10 cells/high-power field (HPF) was detected (**Supplementary Figure S2**). CT findings were graded according to the Lund–Mackay method.

**Table 2:** Characteristics of CRS patients involving in the present study.

	Control	Patients with eCRSwNP	Patients with non-eCRSwNP	Patients with CRSsNP
**Subjects no.**	10	30	28	20
**Male sex**	6(60%)	20 (67%)	13 (46%)	10 (50%)
**Age (y)**	38 ± 7	41 ± 12	44 ± 15	44 ± 15
**Patients with bilateral lesion**	—	14 (47%)	10 (36%)	5 (25%)
**Lund-Mackay score**	**—**	13 ± 4	9 ± 3	6 ± 4
**Eosinophils in PB (10^9^/L)**	0.07 ± 0.04	0.39 ± 0.23	0.10 ± 0.10	0.11 ± 0.12
**Patients with asthma**	0 (0%)	2 (7%)	0 (0%)	0 (0%)
**Patients with atopy**	2 (20%)	10(33%)	7(25%)	5 (25%)
**Patients with aspirin intolerance**	0 (0%)	2(7%)	0 (0%)	1 (5%)

For continuous variables, results are expressed as mean ± standard deviation. Categorical variables are summarized by using percentages. Subjects no., subject number; eosinophils in PB, eosinophils in blood; CRSwNP, chronic rhinosinusitis with nasal polyps; CRSsNP, chronic rhinosinusitis without nasal polyps.

Exclusion criteria for CRS patients: patients treated with oral antibiotics, antileukotrienes, systemic corticosteroids; with an upper respiratory tract infection 4 weeks preceding the operation; who were pregnant; with immune disorders or carcinoids, such as inverting papilloma, or malignancies, such as nasopharyngeal carcinoma were excluded. We also excluded subjects with CRS due to fungal sinusitis, cystic fibrosis, primary ciliary dyskinesia, vasculitis, or other specific causes.

### Immunohistochemistry analysis

Mucosal tissues from patients with CRS were obtained from nasal polyps or the uncinate process. The tissues were immediately fixed in 10% formalin, embedded in paraffin, and cut into thin sections. The sections were stained with hematoxylin and eosin (H&E) (BL735A, Beijing, China) to differentiate the CRS into eosinophilic phenotypes. eCRS was defined histologically as an elevated tissue eosinophil count >10 cells/HPF. Representative H&E-stained images of the non-eCRS and eCRS groups are shown in **Supplementary Figure S2**. The number of eosinophils beneath the epithelial surface per HPF (x400) was quantified by two independent researchers. Five fields were selected randomly.

For expression analysis of Gαi1, Gαi3, IgE, and tryptase, formalin-fixed and paraffin-embedded nasal biopsies were cut into 4 μm thick sections and deparaffinized. Deparaffinized sections were heated in sodium citrate buffer (pH 6.0) for antigen retrieval. After inhibiting endogenous peroxidase with 3% hydrogen peroxide and blocking with 3% bovine serum albumin, the sections were incubated overnight at 4°C in the presence of anti-Gαi1, anti-Gαi3, anti-IgE, and anti-tryptase antibodies. Each section was incubated with horseradish-peroxidase-conjugated secondary mouse or rabbit antibodies for 50 min. After washing, the sections were incubated with 3, 3’-diaminobenzidine tetrachloride, and then immediately in tap water after color development. The sections were counterstained with hematoxylin and mounted with dibutyl phthalate xylene. They were then examined blindly with no awareness of the clinical data using an XSP-C204 microscope (Chongqing, China). The average number of IgE^+^ and tryptase^+^ cells, and the mean optical densities (MODs) of Gαi1 and Gαi3 were determined from five randomly chosen HPFs (x400) using ImageJ (national Institutes of Health, Bethesda, MS, USA) and used to calculate the expression level (20).

### Western blotting

Cells or tissues were lysed with lysis buffer, and the supernatant was collected after centrifugation. Protein concentrations of the samples were determined using a BCA protein quantification kit (Thermo Fisher, USA) on a microplate reader (TECAN, Switzerland). Protein samples were diluted in phosphate buffer saline (PBS) to adjust the concentrations, and 4 μL of Sample Buffer was added. The samples were heated in a metal bath at 95°C for 10 minutes. After preparing the gels, 20 μL or 40 μL of the protein samples were loaded into the wells of an SDS-PAGE gel, with 2.5 μL of Marker added to each side of the sample groups. The electrophoresis was performed at 85 V until the Marker bands separated, then increased to 110 V until the blue front reached the bottom. After transferring the membrane, it was blocked by soaking in PBST containing 10% non-fat dry milk at room temperature for 30 minutes. The primary antibody was diluted in PBST according to the specified ratio and incubated overnight at 4°C on a shaking platform. The following day, the membrane was washed with PBST three times for 10 minutes each. After adding the corresponding secondary antibody, it was incubated at room temperature for 2 hours. Finally, excess antibodies were washed away with PBST three times for 10 minutes each. The membrane was placed in a developing chamber, and ECL/Super ECL was added for darkroom exposure and imaging. Uncropped western blot images are shown in **Supplementary Figure S5**.

We collected five nasal tissue samples from each group for immunoblotting experiments (**Supplementary Figure S5A**). ImageJ was utilized to measure the bands of the target proteins after imaging, and relative expression values were calculated. Blot quantification was performed based on five blot data, and data from all experiments were pooled to calculate the mean ± standard deviation (SD).

### Mouse embryonic fibroblasts culture

Primary MEFs were a gift from M. J. Tevethia (Hershey, PA, USA). As previously reported (9, 16, 21, 22), wild-type (WT); Gαi1, Gαi2, or Gαi3 single-knockout (SKO); and Gαi1 and Gαi3 double-knockout (DKO) MEFs were derived from WT; Gαi1, Gαi2, or Gαi3 single-knockout; and Gαi1 and Gαi3 double-knockout mouse E14.5 (embryonic day 14.5) embryos. MEFs (5 × 10^5^–10 × 10^5^ cells) were then immortalized by transfection with the total SV40 genome (plasmid pSV40WT) and subcultured several times in Dulbecco’s modified Eagle medium supplemented with 10% FBS and 1% antibiotics (penicillin and streptomycin). WT and Gab1-KO MEFs were used as described previously (23). The MEFs were starved overnight in 0.5% FBS and incubated for 30 min in warm PBS before treatment.

### EoL-1 cell culture

EoL-1 cells, a human eosinophilic cell line generated from a patient with acute myeloid leukemia following hypereosinophilic syndrome, were purchased from Honsun Biological Technology (Shanghai, China). The cells were cultured in RPMI-1640 medium supplemented with 10% FBS and 1% antibiotics (penicillin and streptomycin) in a humidified atmosphere at 37°C with 5% CO_2_. Fresh medium was added every 2–3 d. Cultures were maintained at cell concentrations between 1 × 10^5^ and 1 × 10^6^ viable cells/mL.

### Gαi1/3 shRNA lentivirus transfection

EoL-1 cells were seeded into six-well tissue culture plates at 1 × 10^5^ cells per well and transfected with human Gαi1 short hairpin (sh)RNA lentivirus (multiplicity of infection [MOI] = 20) and/or the human Gαi3 shRNA lentivirus (MOI = 20) (9, 16). After 24 h, EoL-1 cells with the target shRNA were selected by puromycin (1.0 μg/mL). The culture medium was replaced with fresh puromycin-containing culture medium every 2 d, until resistant colonies were formed (6–7 d). In stable cells, Gαi1/3 knockdown was verified by western blotting and quantitative PCR (qPCR). Control EoL-1 cells were infected with scramble nonsense shRNA lentiviral particles. A previously reported protocol was used to transfect WT MEFs with Gαi1 and Gαi3 shRNAs (16, 21).

### qPCR

Cells or tissues were lysed with Trizol for 5 minutes, followed by the addition of chloroform and shaking. After standing for 10 minutes, centrifugation was performed. The upper aqueous phase was collected and mixed with an equal volume of isopropanol, followed by centrifugation to obtain RNA precipitation. The RNA was washed with 75% ethanol and then dried. RNA concentration was measured using a micro-spectrophotometer (Thermo Fisher, USA), and RNA samples with an OD 260/280 ratio of 1.9-2.1 were used for subsequent experiments. Reverse transcription was performed using a reverse transcriptase kit (Vazyme, China) to generate cDNA. PCR primers were dissolved in DEPC water to a concentration of 100 μM. The SYBR qPCR Master Mix protocol (Vazyme, China) was followed to set up the real-time quantitative PCR system in a 96-well plate. The real-time PCR reaction program was set on a fluorescent quantitative PCR instrument (Applied Biosystems, USA) as follows: Phase 1: 95°C for 30 seconds; Phase 2: 40 cycles of 95°C for 3-10 seconds and 60°C for 10-30 seconds; Phase 3: 95°C for 15 seconds, 60°C for 60 seconds, and 95°C for 15 seconds.

### EdU/DAPI staining

Cells (1 × 10^6^) were seeded in 6-well plates and treated with drugs for 1 day. After collecting the cells through centrifugation, they were resuspended in complete medium containing 0.02% EdU solution (Reagent A) from the EdU kit (R11053-9, Guangzhou, China) and transferred back to 6-well plates for 2 hours in an incubator. Following PBS washes, the cells were fixed in 4% PFA for 30 minutes. A 2 mg/mL Glycine solution was prepared in ddH2O and used to incubate the cells for 5 minutes on a shaking platform. After washing, cells were treated with 0.5% Triton X-100 (Sigma-Aldrich, USA) for 10 minutes. Next, 500 μL of Apollo staining solution (containing 469 μL ddH2O, 25 μL Reagent B, 5 μL Reagent C, 1.5 μL Reagent D, and 5 mg Reagent E) was added and incubated at room temperature in the dark for 30 minutes. Cells were washed three times with 1 mL of 0.5% Triton X-100, each wash lasting 10 minutes. After centrifugation and removal of the supernatant, 500 μL of ddH2O was added, mixed with a 100:1 dilution of Reagent F, and incubated in the dark at room temperature for 30 minutes. Cells were resuspended in PBS and transferred to 24-well plates, with images captured 24 hours later using a fluorescence inverted microscope (Carl Zeiss, Germany).

### Co-immunoprecipitation

After drug treatment, cells were lysed in a culture dish. The lysate was centrifuged, and the supernatant was collected. Twenty μL of Agarose A/G beads (sc-2003, CA, USA) were added, and the sample was rotated at 4°C for 10 minutes to pre-wash the lysate. The total protein was diluted tenfold in PBS, and the concentration was determined using the BCA method, adjusting the protein to 1 μg/μL with PBS. The primary antibody was added and incubated overnight at 4°C while shaking. Another 20 μL of Agarose A/G beads was added, followed by overnight rotation at 4°C. The mixture was centrifuged at 4°C for 5 minutes to collect the precipitate, which was washed three times with PBS. After adding 60 μL of Sample Buffer, the mixture was boiled for 10 minutes to separate the beads. The solution was centrifuged at 8000 rpm for 60 seconds at 4°C, and the supernatant was saved at -20°C for subsequent western blotting.

### Immunofluorescence staining

Slides were immersed in 5% ethanol for 5 minutes, then placed in an incubator to dry before being coated with poly-L-lysine for 2 hours. Excess liquid was discarded, and the slides were washed three times with PBS. The processed cells were added to the slides, followed by a PBS wash. Cells were fixed with 4% PFA for 15 minutes and subsequently washed with PBS. The slides were permeabilized in PBS containing 0.25% Triton X-100 at room temperature for 20 minutes, followed by three PBS washes. The slides were blocked with PBS containing 5% goat serum for 30 minutes. After discarding the excess serum, the appropriate dilution of primary antibody was added and incubated overnight at 4°C. Fluorescently labeled secondary antibody was prepared and added to the cells, incubated at room temperature in the dark for 1 hour. Finally, coverslips were mounted with an anti-fade mounting medium and stored at 4°C in the dark, with images captured using a laser confocal microscope (Nikon, Japan).

### Mice

All mice used in the experiments had a C57BL/6 background. The generation of Gαi1/3 DKO mice using the CRISPR-Cas9 method has been described previously (16). WT C57BL/6 and Gαi1/3-DKO C57BL/6 mice were bred and housed at our facilities. All mice were kept under specific pathogen-free conditions, and all experiments were conducted according to the institutional regulations.

### Establishment of murine model of eCRSwNP

The mice were divided into one control group and four experimental groups. Each group included eight mice; three mice were used for histological analyses and five mice were used for mRNA analyses. All mice were used for serum and nasal lavage analyses. During the modeling process, two mice died in the OVA group and one mouse died in the DKO group. The control group was treated with PBS only. The experimental groups were treated as follows: instillation with 10 ng of SEB (SEB group), instillation with OVA only (OVA group), instillation with OVA plus 10 ng SEB in DKO mice (DKO group), and instillation with OVA plus 10 ng SEB in WT mice (CRS group).

Mice in the CRS group were systemically sensitized with 25 g of OVA dissolved in 300 mL of PBS in the presence of 2 mg of aluminum hydroxide gel as an adjuvant by intraperitoneal injection on days 1 and 6, followed by daily intranasal instillation with 40 μL of 3% OVA from days 13 to 20. Thereafter, continuous local stimulation was maintained in the same manner three times per week for 12 consecutive weeks. In addition to 3% OVA, the mice were challenged weekly with SEB (10 ng) 5–12 weeks after OVA instillation.

Twenty-four hours after the final nasal challenge, mice were euthanized and decapitated. The nasal lavage fluid, serum, and tissue samples were collected (24). Serum and nasal lavage fluid were analyzed in eight mice. Three mice were prepared for histological examination and the nasal mucosa of the remaining five mice was used for qPCR (**Supplementary Figure S1**). One mouse in the DKO group and two mice in the OVA group died during the development of inflammation.

For sample preparation, the mice were decapitated and the lower jaw and tongue were removed. Using the hard palate as a guide, a large scalpel was used to remove the snout with a transverse cut behind the back molars. After removing the skin and excess soft tissue, the external nares were flushed with PBS to remove the blood. The protocol was approved by the Ethics Committee of Soochow University (ECSU) (No. SUDA20230727A06).

### Histological analysis

The heads of the mice were immediately fixed in 4% PFA (FD9680, Hangzhou, China) and decalcified in an EDTA decalcifying solution (RE4321, Wuhan, China) for 15 d. Tissues were dehydrated and processed using standard paraffin-embedding procedures. A coronal section 3 mm posterior to the mouse eye orbit was selected for evaluation. To characterize inflammatory changes, several stains were performed, including H&E staining (BL735A, Beijing, China) for inflammatory cells, Sirius red staining (RE4103, Wuhan, China) for eosinophils, and toluidine blue staining (RE4101, Wuhan, China) for MCs. Refine the labeling of slices, conduct microscopic examination, register them, and take full-scanner photographs (Olympus, China). The numbers of polyp-like lesions and eosinophils were counted in HPFs. Polyp-like lesions were defined as distinct mucosal bulges with eosinophilic infiltration and/or microcavity formation (24).

H&E staining:Deparaffinize the paraffin sections and wash with tap water for 1-2 minutes. Stain with hematoxylin solution for 3-5 minutes, then rinse away the hematoxylin with running water. Stain with eosin solution for another 3-5 minutes, and wash off any excess eosin with water. Dehydrate the sections through a series of graded alcohols, treat with xylene for clearing, and finally mount the slides.

Sirius red staining:Fix the sections in 4% PFA for 30 minutes, then wash with distilled water three times, each for 2 minutes. Stain with Sirius Red solution for 1 hour, followed by two water washes. Quickly wash twice with 0.5% acetic acid solution. Stain the cell nuclei with hematoxylin for 8 minutes and rinse in tap water for 10 minutes. Dehydrate through graded ethanol and clear in xylene.

Toluidine blue staining: Deparaffinize the sections to water, and rinse in a series of ethanol solutions for 1 minute each, followed by a wash in tap water for 2 minutes. Stain with Toluidine Blue O for 15-30 minutes. Briefly wash in tap water, then differentiate with TBO differentiation solution until the cell nuclei and granules are clearly visible. After a slight rinse in tap water, blot excess moisture on filter paper, dry, then immerse in 95% ethanol for 1 minute, followed by two immersions in absolute ethanol, each for 2 minutes. Finally, clear in xylene and mount with neutral gum.

### ELISA

Serum samples from mouse orbits were stored at -70°C for subsequent measurement of IgE. Quantitative assessments of total and OVA-specific IgE levels in the serum were performed using ELISAs. The sensitivities of the total IgE and OVA-specific IgE ELISAs were 0.78 and 20.7 ng/mL.

To collect nasal lavage fluid, 400 μL of PBS was flushed through the nasal cavity from the posterior choanae to the anterior nostrils using a pipette tip after the lower jaw was resected, and approximately 400 μL of nasal lavage fluid was collected. The supernatants were stored at -70°C for the analysis of chemokines and cytokines, including eotaxin (CCL11), IL-4, IL-5, and IL-13, using ELISAs. The lower detection limits of the ELISA kits were 0.73 pg/mL for CCL11, 3.9 pg/mL for IL-4, 7.8 pg/mL for IL-5, and 7.8 pg/mL for IL-13. All procedures were performed according to manufacturer’s instructions as follows.

First, set up standard wells, blank wells, and sample wells. Add a specific volume of standard sample to the wells. Add the prepared biotinylated antibody working solution to each well. Incubate at 37°C for 45 minutes, following the specific time outlined in the instructions. Soak the wells in wash buffer for one minute, then remove the liquid, tap dry, and repeat the washing process three more times. Next, add the enzyme conjugate working solution to each well, and incubate at 37°C for 30 minutes, followed by five washings. Then, add the substrate solution, incubating at 37°C in the dark for 15 minutes. Preheat the microplate reader 15 minutes in advance. Finally, add the stop solution to each well and measure the optical density (OD) at a wavelength of 450 nm using the microplate reader (TECAN, Switzerland).

### Bioinformatics analysis

Gene expression profiles of the GSE10406 (https://www.ncbi.nlm.nih.gov/geo/query/acc.cgi?acc=GSE10406) and GSE36830 (https://www.ncbi.nlm.nih.gov/geo/query/acc.cgi) datasets were downloaded from the Gene Expression Omnibus (GEO) database. The GSE10406 dataset comprised 18 samples, including 9 CRS samples and 9 normal nasal tissue samples. The GSE36830 dataset comprised 24 samples, including 19 CRS samples and 6 normal nasal tissue samples. Original data for expression levels were downloaded.

### Statistical analyses

Except for multivariate regression analyses, which were performed using SPSS 29.0 software (SPSS, Chicago, IL, USA), all data were analyzed using GraphPad Prism 6 software (GraphPad, San Diego, CA, USA). The normality of the variables was evaluated using the Shapiro-Wilk test. Student’s unpaired t-tests were performed for two-group comparisons of data with a normal distribution. Multiple group comparison was performed by one-way analysis of variance (ANOVA) with *post hoc* Bonferroni test (data were all with normal distribution). If the data does not follow a normal distribution, the Wilcoxon rank-sum test is used for comparisons between two groups, while the Kruskal-Wallis rank-sum test is employed for comparisons among multiple groups. Bonferroni correction is applied for *post hoc* comparisons. Interactions between variables were assessed using Pearson’s or Spearman’s correlation tests for normally and non-normally distributed variables, respectively. All data are presented as the mean ± standard deviation. p < 0.05 was considered statistically significant.

## Results

### Increased nasal mucosal Gαi1/3 expression in patients with CRS, especially those with eCRS

**Figure 1:**
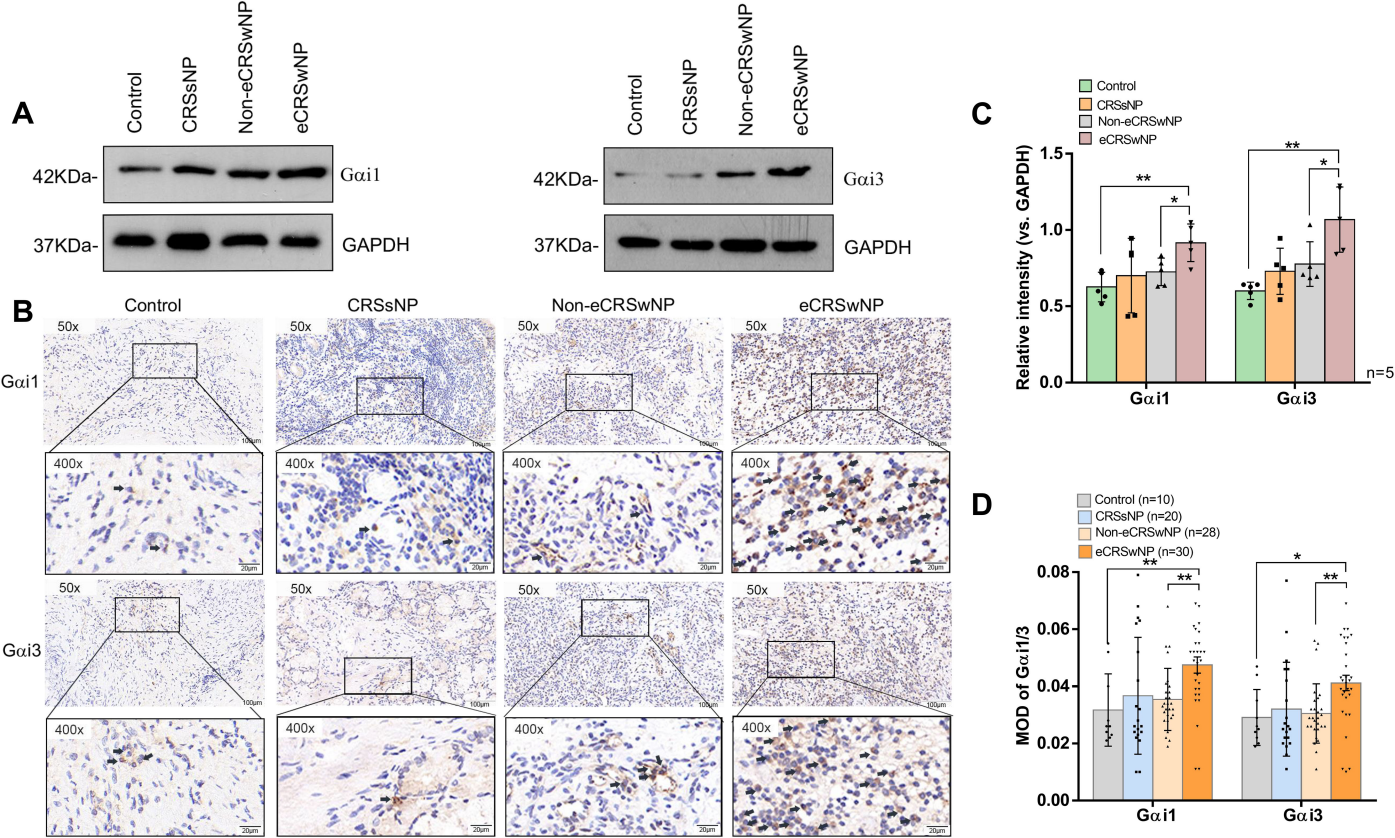
The expression of Gαi1/3 are upregulated in the nasal tissues of CRS patients. Expression of Gαi1, Gαi3, and GAPDH in diseased nasal mucosa in patients with CRSsNP and those with eCRSwNP and non-eCRSwNP **(A)** was tested by western blotting. Gαi1/3 IHC of patients with CRSsNP and those with eCRSwNP and non-eCRSwNP **(B)**. The indicator proteins in western blotting were quantified **(C)**. Bar = 100 μm. Insets show a higher magnification of the outlined area. Quantitative analysis of the expression of Gαi1/3 in IHC was conducted under a microscope **(D)**. eCRSwNP, eosinophilic CRSwNP; non-eCRSwNP, non-eosinophilic CRSwNP; MOD, mean optical density. *P <0.05 and **P <0.01.

We first determined the Gαi1/3 expression levels in patients with CRS by western blotting. We collected 5 cases each for western blotting testing in our respective groups and found that the expression levels of Gαi1/3 proteins increased in CRS patients compared to the control group (**Figures 1A, C**). We then focused on the role of Gαi1/3 in the development of nasal polyps. There were no significant differences in Gαi1/3 levels between CRSsNP patients and CRSwNP patients (**Figure 1C**). Among the different types of CRS, eCRSwNP demonstrated the highest levels of local Gαi1/3 expression (**Figure 1C**). Of note, nasal mucosal Gαi1 (0.92 ± 0.12 vs. 0.73 ± 0.09, *p* = 0.0233) and Gαi3 (1.07 ± 0.21 vs. 0.78 ± 0.15, *p* = 0.0360) expression levels were higher in patients with eCRSwNP than in those with non-eCRSwNP (**Figure 1C**).

We found that Gαi1/3 expression levels were higher in CRS patients than in the control group by western blotting(0.63 ± 0.10 vs. 0.78 ± 0.18, p = 0.0306; 0.60 ± 0.06 vs. 0.86 ± 0.22, p = 0.0220). Therefore, we collected more cases for immunohistochemical analyses to explore the potential differences in Gαi1/3 expression levels between the CRS subtypes. We found that the expression levels of Gαi1 (0.05 ± 0.02 vs. 0.03 ± 0.01, *p* = 0.0075, **Figure 1D**) and Gαi3 (0.04 ± 0.02 vs. 0.03 ± 0.01, *p* = 0.0155, **Figure 1D**) proteins increased in eCRSwNP patients compared to the control group (**Figures 1B, D**). Consistent with the western blotting results, IHC results (**Figure 1B**) showed a significant increase in Gαi1 (0.05 ± 0.02 vs. 0.04 ± 0.01, *p* = 0.0011, **Figure 1D**) and Gαi3 (0.04 ± 0.02 vs. 0.03 ± 0.01, *p* = 0.0025, **Figure 1D**) expression levels in nasal tissues of patients with eCRSwNP, whereas the expression levels of these two proteins were relatively low in patients with non-eCRSwNP.

### High nasal tissue Gαi1/3 levels are linked to high disease severity and allergic conditions of CRS patients

**Figure 2:**
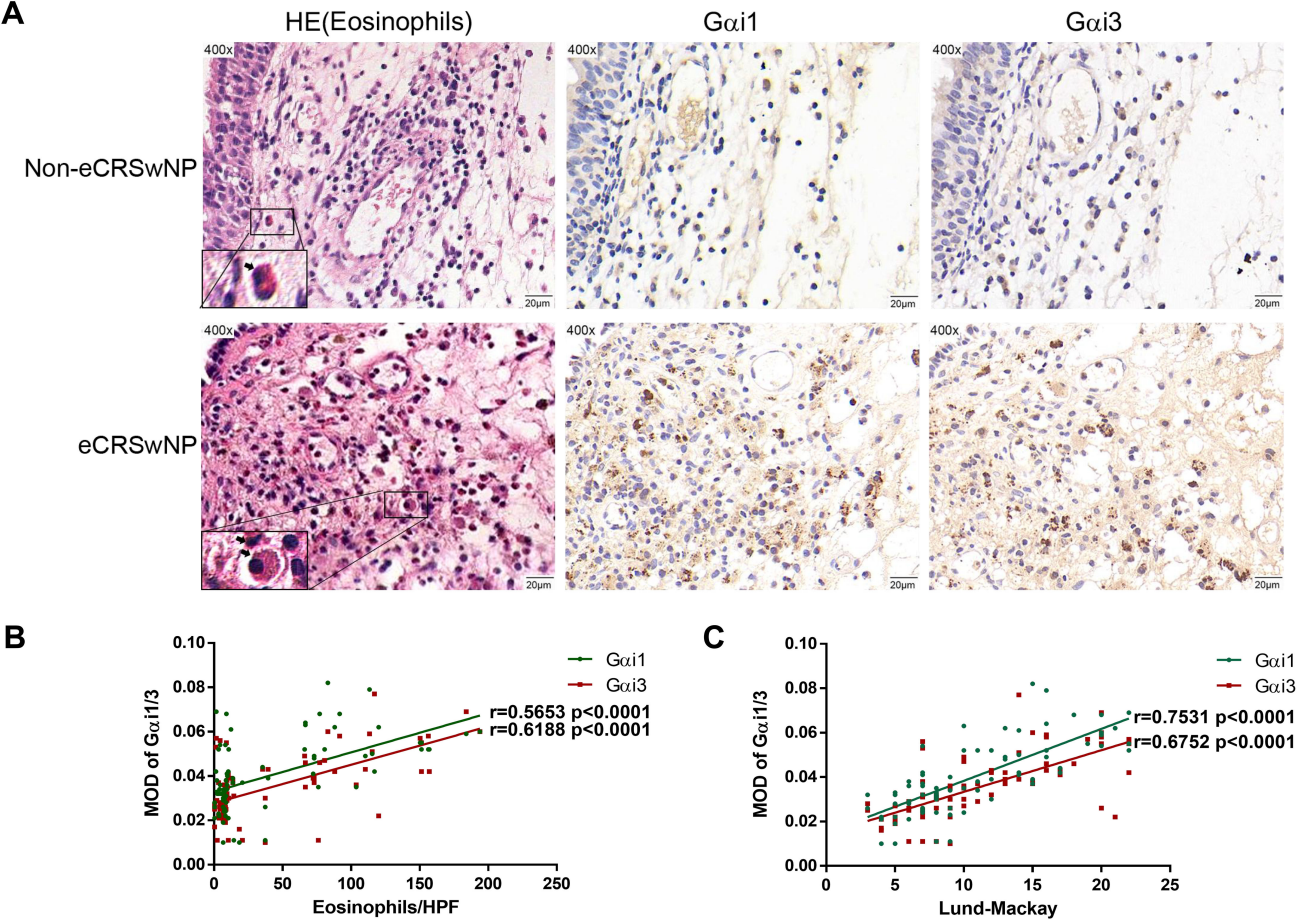
Gαi1/3 play a role in allergic inflammatory response of eCRS. Nasal mucosa tissues were also fixed and subjected to hematoxylin-eosin staining, the image on the right shows IHC in the same region **(A)**. Bar = 20 μm. Insets show a higher magnification of the outlined area. Arrows denote eosinophils. Correlations of Gαi1/3 levels with numbers of eosinophils and Lund-Mackay CT scores in patients **(B, C)**. eCRSwNP, eosinophilic CRSwNP; non-eCRSwNP, non-eosinophilic CRSwNP; MOD, mean optical density.

The histological analysis results showed that the number of eosinophils in the same local tissue area of eCRSwNP patients and the expression levels of Gαi1/3 both increased (**Figure 2A**), and positive correlations between Gαi1 or Gαi3 levels and eosinophil counts in eCRSwNP were also observed (r = 0.5653, p < 0.0001; r = 0.6188, p < 0.0001, **Figure 2B**). Eosinophilic upregulation in CRS has been associated with higher CT and endoscopic scores, as it is more likely to be observed in eCRS. We found that Gαi1/3 expression levels were positively associated with Lund-Mackay CT scores (r = 0.7531, *p* < 0.0001; r = 0.6752, *p* < 0.0001, **Figure 2C**).

**Figure 3:**
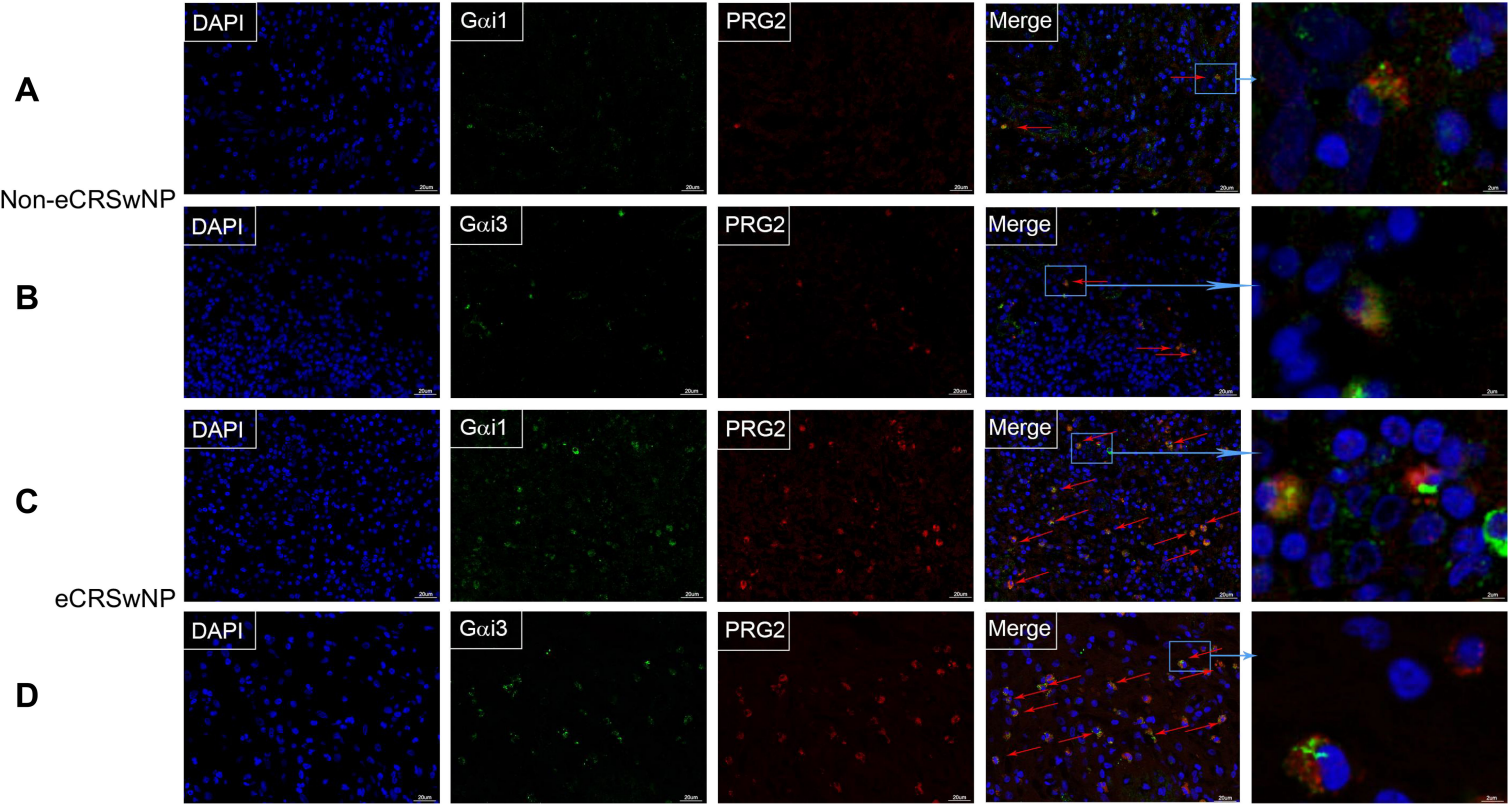
The expression of Gαi1/3 increases in parallel with eosinophilic infiltration in CRS. Nasal mucosal tissue from CRS patients, which was then fixed, sectioned, and subjected to dual immunofluorescence staining for Gαi1/3 and eosinophils. In non-eCRSwNP patients, dual staining revealed the presence of Gαi1 **(A)** and Gαi3 **(B)** alongside eosinophils. Similarly, in eCRSwNP patients, Gαi1 **(C)** and Gαi3 **(D)** were also observed in conjunction with eosinophils. Arrows indicate co-stained cells, bar = 20 μm. The far-right column displays magnified images of representative co-stained cells, bar = 2 μm.

Eosinophilic infiltration of nasal mucosal tissue is a key immunological feature in the pathogenesis of eCRS. To further investigate the role of Gαi1/3 in eCRS, we performed immunofluorescence staining on biopsy samples from patients with non-eCRS and eCRS (**Figure 3**). Our findings revealed co-expression of Gαi1/3 with eosinophils in the diseased nasal tissues. Moreover, consistent with the above results, the expression of Gαi1/3 significantly increased in the nasal tissues of eCRSwNP patients compared to non-eCRSwNP patients, correlating with the heightened eosinophilic infiltration (**Figure 3**).

Moreover, the local expression of Gαi1/3 in nasal mucosa was upregulated with

an increase in the peripheral blood eosinophil count, which can reflect an allergic inflammatory response (r = 0.3651, *p* = 0.0010; r = 0.4629, *p* < 0.0001, **Table 3**). We detected the expression of these two proteins in patients with CRS with and without atopy. As a result, nasal mucosal Gαi1 (0.05 ± 0.02 vs. 0.04 ± 0.02, *p* = 0.0251) and Gαi3 (0.04 ± 0.01 vs. 0.03 ± 0.01, *p* = 0.0030) expression was higher in patients with atopy than that in patients without atopy (**Table 4**). In multivariable regression analyses, we found that atopy had a significant impact on Gαi1 (*p* = 0.035) and Gαi3 (*p* = 0.003) expression levels in patients with CRS (**Tables 5**, **6**). Meanwhile, neither aspirin intolerance (*p* = 0.523; *p* = 0.665) nor asthma (*p* = 0.309; *p* = 0.248) had a significant effect on the protein levels of Gαi1 and Gαi3 (**Tables 5**, **6**).

**Table 3:** Correlation analysis of Gαi1 and Gαi3 expression and clinical parameters in CRS patients.

Parameter1	Parameter2	R value	P value
Lund-Mackay score	Tissue eosinophil count/HPF	0.654	**<0.0001**
MOD of Gαi1	Lund-Mackay score	0.753	**<0.0001**
MOD of Gαi3	Lund-Mackay score	0.675	**<0.0001**
MOD of Gαi1	Blood eosinophil count (10^9^/L)	0.365	**0.001**
MOD of Gαi3	Blood eosinophil count (10^9^/L)	0.463	**<0.0001**
MOD of Gαi1	Blood basophil count (10^9^/L)	0.150	0.191
MOD of Gαi3	Blood basophil count (10^9^/L)	0.181	0.112
MOD of Gαi1	Blood neutrophil count (10^9^/L)	0.064	0.575
MOD of Gαi3	Blood neutrophil count (10^9^/L)	0.125	0.276

Boldface indicates P < 0.05.

**Table 4:** Unpaired T-test between patients.

	MOD of Gαi1	MOD of Gαi3
**Patients with bilateral lesion**	0.041 ± 0.013	0.035 ± 0.012
**Patients with unilateral lesion**	0.040 ± 0.018	0.035 ± 0.016
**P value**	0.916	0.973
**Patients with atopy**	0.047 ± 0.015	0.043 ± 0.014
**Patients without atopy**	0.038 ± 0.016	0.032 ± 0.014
**P value**	**0.025**	**0.003**
**Patients with aspirin intolerance**	0.046 ± 0.014	0.039 ± 0.004
**Patients without aspirin intolerance**	0.040 ± 0.016	0.035 ± 0.015
**P value**	0.539	0.248

Values were expressed as mean ± standard deviation. Boldface indicates P < 0.05.

**Table 5:** Multivariate analysis of associations between NERD, asthma, atopy factors, and protein expression level of Gαi1 in nasal tissue.

Variable quantity	Regression coefficient	Standard error	Standardized regression coefficient	T value	P value
**Asthma**	0.012	0.011	0.115	1.025	0.309
**Atopy**	0.009	0.004	0.240	2.143	**0.035**
**NERD**	0.006	0.009	0.072	0.642	0.523

NERD, non-steroidal anti-inflammatory drugs-exacerbated respiratory disease. R^2^ = 0.08. Boldface indicates P < 0.05.

**Table 6:** Multivariate analysis of associations between NERD, asthma, atopy factors, and protein expression level of Gαi3 in nasal tissue.

Variable quantity	Regression coefficient	Standard error	Standardized regression coefficient	T value	P value
**Asthma**	0.012	0.010	0.126	1.163	0.248
**Atopy**	0.011	0.003	0.337	3.108	**0.003**
**NERD**	0.004	0.008	0.047	0.435	0.665

NERD, non-steroidal anti-inflammatory drugs-exacerbated respiratory disease. R^2^ = 0.138. Boldface indicates P < 0.05.

### Increased IgE levels and MCs infiltration in eCRS patients

Local infiltration of nonspecific IgE^+^ and MCs in the nasal mucosa is correlated with clinical severity and histopathological factors in eCRS, which are pathophysiologically driven by eosinophilic infiltration (5, 25, 26). Consistent with previous research, we found that the numbers of tryptase^+^ (17.33 ± 6.12 vs. 8.99 ± 4.26, *p* < 0.0001, **Supplementary Figure S3B**) and IgE^+^ cells (19.62 ± 5.68 vs. 8.43 ± 4.40, *p* < 0.0001, **Supplementary Figure S3B**), in nasal mucosal tissues were increased in patients with eCRSwNP than those with non-eCRSwNP, as detected by using immunostaining (**Supplementary Figure S3A**). This analysis demonstrated increased numbers of IgE^+^ and tryptase^+^ cells in the tissues of patients with eCRSwNP compared to those with CRSsNP (**Supplementary Figure S3B**). Furthermore, we observed significant associations between the number of tryptase^+^ and IgE^+^ cells in our CRS patients (r = 0.8344, *p* < 0.0001; **Supplementary Figure S3C**). The numbers of tryptase^+^ and IgE^+^ cells were also associated with Lund-Mackay CT scores (r = 0.6602, *p* < 0.0001, **Supplementary Figure S3D**; r = 0.6562, *p* < 0.0001, **Supplementary Figure S3E**).

### Gαi1/3 are required for IL-5-induced Akt-mTOR and Erk activation in MEFs

Based on the above mentioned findings, high Gαi1/3 expression levels were significantly correlated with the number of eosinophils in nasal tissue and more positively correlated with eCRS type in CRS patients. IL-5 is a major cytokine correlated with eosinophil development, activation, and survival. Thus, we first investigated the potential role of Gαi1/3 in IL-5-induced activation in tool cells, i.e., MEFs.

**Figure 4:**
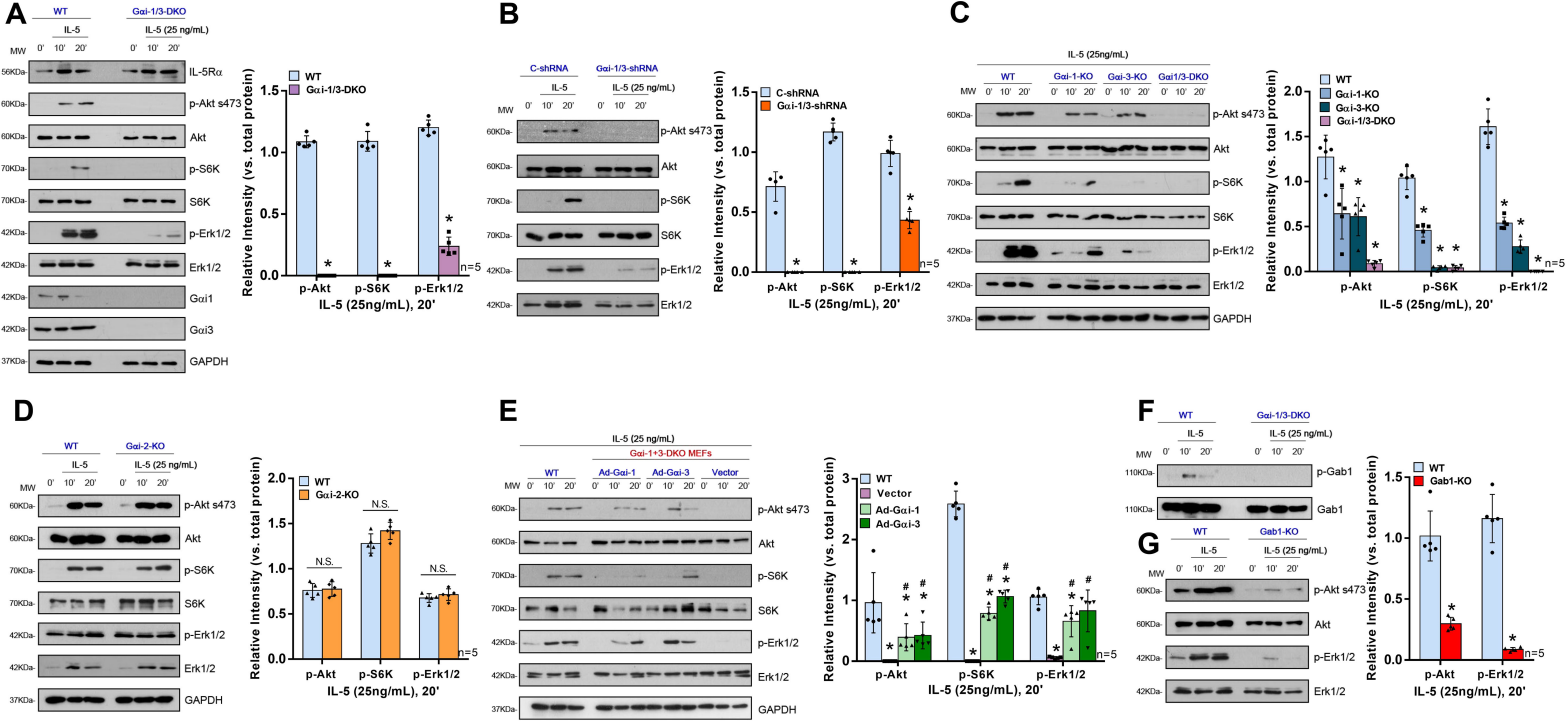
Gαi1/3 are required for the activation of Akt and Erk by IL-5 in MEFs. WT, Gαi1/3 double knockout **(A, F)**, Gαi1, Gαi2 or Gαi3 single knockout **(C, D)** mouse embryonic fibroblasts were treated with IL-5 (25 ng/mL) for applied time, tested by Western blotting of listed proteins **(A, C, D, F)**; DKO MEFs were transiently transfected with the adenovirus Gαi1 construct (“Ad-Gαi-1”), the adenovirus Gαi3 construct (“Ad-Gαi-3”) or the empty vector, IL-5-induced signaling was tested similarly **(E)**. Stable WT MEFs with the scramble control shRNA (“C-shRNA”), Gαi1 shRNA and Gαi3 shRNA (“Gαi-1/3-shRNA”), were treated with IL-5 (25 ng/mL) for applied time, and were tested by Western blotting of listed proteins **(B)**. WT or Gab1 KO MEFs were treated with IL-5 (25 ng/mL) for applied time, tested by Western blotting of listed proteins **(G)**. Quantification was performed from five replicate blot data. Data were expressed as mean ± SD. WT, wild-type; DKO, double knockout; SKO, single knockout; MEFs, mouse embryonic fibroblasts; NS, no significant difference. *P <0.05 vs. WT MEFs **(A, C, E, G)**. # P <0.05 vs. “Vector” **(E)**. *P <0.05 **(B)**.

Initially, we utilized MEFs derived from WT or Gαi1- and Gαi3-deficient (Gαi1/3 DKO) mouse embryos. Baseline total IL-5Rα expression levels were similar between the WT and DKO MEFs (**Figure 4A**). We first tested the requirement of Gαi proteins for the activation of Akt, S6K, and Erk1/2 (Thr202/Tyr204) by IL-5. As shown in **Figure 4**, IL-5-induced phosphorylation of Akt, S6K, and Erk1/2 was significantly inhibited in Gαi1/3-DKO MEFs (0.00 ± 0.00 vs. 1.09 ± 0.05, *p* < 0.0001; 0.00 ± 0.00 vs. 1.09 ± 0.08, *p* < 0.0001; and 0.24 ± 0.08 vs. 1.20 ± 0.06, *p* < 0.0001, respectively; **Figure 4A**) and in WT MEFs transfected with Gαi1/3-shRNA (0.00 ± 0.00 vs. 0.71 ± 0.12, *p* = 0.0002; 0.00 ± 0.00 vs. 1.17 ± 0.07, *p* < 0.0001; 0.43 ± 0.07 vs. 0.99 ± 0.11, *p* < 0.0001, respectively; **Figure 4B**) compared with WT MEFs. Meanwhile, in contrast to Gαi1 or Gαi3 SKO, Gαi2 SKO failed to significantly affect IL-5-induced signaling in MEFs (Akt: 0.78 ± 0.08 vs. 0.76 ± 0.08, *p* = 0.7683; S6K: 1.42 ± 0.10 vs. 1.28 ± 0.11, *p* = 0.0599; Erk1/2: 0.72 ± 0.06 vs. 0.68 ± 0.05, *p* = 0.3298; **Figure 4D**). These data indicated the selective requirement for Gαi1/3 proteins in the activation of Akt and Erk1/2 signaling in response to IL-5 stimulation. SKO of Gαi1 or Gαi3 in MEFs resulted in partial inhibition of Akt, S6K, and Erk1/2 phosphorylation in response to IL-5 (**Figure 4C**). It should be noted that Gαi3 knockout in MEFs resulted in a larger reduction in IL-5 signaling compared to Gαi1 knockout (**Figure 4C**).

To further confirm that loss of *Gαi1*/*3* genes was responsible for IL-5-induced activation failure in MEFs, rescue experiments were performed in DKO MEFs using an adenovirus Gαi1 construct (“Ad-Gαi1”, no Tag) or Gαi3 construct (“Ad-Gαi3”, no Tag) to exogenously express the Gαi1/3 proteins. After re-expression of Gαi1 or Gαi3, IL-5-induced Akt (0.39 ± 0.22 vs. 0.00 ± 0.00, *p* = 0.0169; 0.42 ± 0.22 vs. 0.00 ± 0.00, *p* = 0.0125, respectively), S6K (0.79 ± 0.11 vs. 0.00 ± 0.00, *p* < 0.0001; 1.07 ± 0.11 vs. 0.00 ± 0.00, *p* < 0.0001, respectively), and Erk1/2 (0.66 ± 0.26 vs. 0.06 ± 0.02, *p* = 0.0008; 0.82 ± 0.34 vs. 0.06 ± 0.02, *p* = 0.0078) activation were partially restored in DKO MEFs (**Figure 4E**).

### Gαi1/3 knockdown inhibits IL-5-induced STAT5 activation and the differentiation and growth of EoL-1 cells

**Figure 5:**
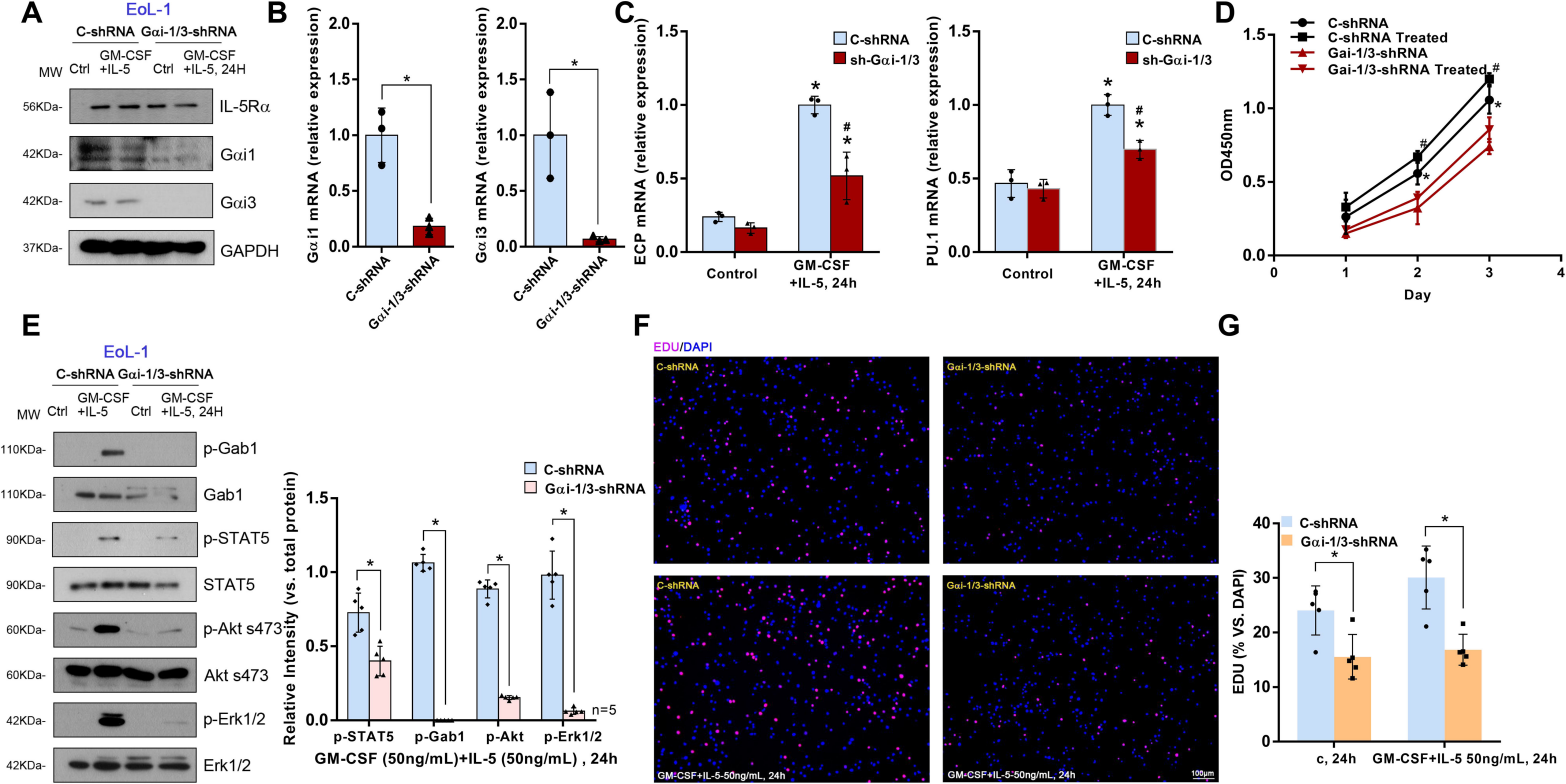
Gαi1/3 knockdown inhibits STAT5 activation, differentiation and growth by IL-5 in EoL-1 cells. Stable EoL-1 cells, expressing the scramble control shRNA (“C-shRNA”), Gαi1 shRNA and Gαi3 shRNA, were treated with GM-CSF (50 ng/mL) and IL-5 (50 ng/mL) for 24 hours, and were tested by Western blotting of listed proteins **(A, E)**. Relative expression of listed genes (24 hours after GM-CSF and IL-5 treatment) was shown **(B, C)**; In each experiment, n=3 (three replicated wells). Cells were further cultured for the times indicated, cell proliferation were tested **(F, G)**, with cell viability tested by CCK-8 assay **(D)**. Bar = 100 μm. Blotting quantification was performed from five replicate blot data, data of all repeated experiments were pulled together to calculate mean ± SD. *P <0.05 **(B, E, G)**. *P <0.05 vs. “Control” treatment in “C-shRNA” EoL-1 cells **(C)**. # P <0.05 vs. GM-CSF and IL-5 treatment in “C-shRNA” EoL-1 cells **(C)**. *P <0.05 vs. “Control” treatment in “Gαi1/3-shRNA” EoL-1 cells **(D)**. #P <0.05 vs. GM-CSF and IL-5 treatment in “Gαi1/3-shRNA” EoL-1 cells **(D)**.

To further investigate the role of Gαi1/3 in IL-5-induced signaling in eosinophils, a human eosinophilic EoL-1 cells were utilized. EoL-1 cells were co-transfected with *Gαi1* and *Gαi3* shRNA lentiviral particles (27), and following selection, stable cells were produced (“shGαi1/3” EoL-1 cells). Cells transfected with lentiviral particles containing a negative control shRNA were used as controls (“C-shRNA” EoL-1 cells). mRNA and protein expression of Gαi1 and Gαi3 were significantly decreased in shGαi1/3 EoL-1 cells (**Figures 5A, B**). Significantly, IL-5 + GM-CSF–induced STAT5 (0.40 ± 0.10 vs. 0.73 ± 0.13, *p* = 0.0023), Akt (0.15 ± 0.01 vs. 0.89 ± 0.06, *p* < 0.0001), and Erk1/2 (0.06 ± 0.02 vs. 0.98 ± 0.16, *p* < 0.0001) phosphorylation was almost completely blocked (**Figure 5E**) by Gαi1/3 silencing in EoL-1 cells. These results indicated that Gαi1 and Gαi3 are required for IL-5 + GM-CSF–induced STAT5 activation in EoL-1 cells.

Thus, we hypothesized that Gαi1 and Gαi3 are necessary for IL-5 + GM-CSF–induced differentiation, including degranulation, of EoL-1 cells. ECP, a basic cationic protein, is released during the degranulation of eosinophils (28) and the transcription factor, PU.1, regulates the differentiation of eosinophils and the transcription of their granule proteins (29). We found that IL-5 and GM-CSF significantly increased the mRNA expression levels of *ECP* (1.00 ± 0.06 vs. 0.24 ± 0.03, *p* = 0.0003) and *PU.1* (1.00 ± 0.07 vs. 0.47 ± 0.10, *p* = 0.0020) in “C-shRNA” EoL-1 cells (**Figure 5C**). However, shRNA-mediated knockdown of Gαi1/3 significantly decreased the mRNA levels of *ECP* (0.52 ± 0.16 vs. 1.00 ± 0.06, *p* = 0.0242) and *PU.1* (0.70 ± 0.06 vs. 1.00 ± 0.07, *p* = 0.0053, **Figure 5C**).

Cell viability was also obviously decreased in Gαi1/3-silenced EoL-1 cells (2 d: 0.32 ± 0.11 vs. 0.56 ± 0.08, *p* = 0.0452, **Figure 5D**). In addition, Gαi1/3 silencing robustly hindered EdU incorporation and decreased the percentage of EdU-positive nuclei in EoL-1 cells, causing significant proliferation inhibition (15.56% ± 4.09% vs. 24.06% ± 4.50%, *p* = 0.0143, **Figures 5F, G**).

### Gαi1 and Gαi3 are required for IL-5-induced IL-5Rα endocytosis and Gab1 recruitment in MEFs and EoL-1 cells

Gab1 is an adaptor protein for Gαi proteins; therefore, we hypothesized that Gαi1/3 may activate downstream signaling pathways in IL-5-induced signaling by mediating Gab1 activation. IL-5 also induced Gab1 phosphorylation in MEFs (**Figure 4F**) and EoL-1 cells (**Figure 5E**). To further investigate the key role of Gab1 in IL-5-induced signaling, we constructed Gab1-KO MEFs. In Gab1-KO MEFs, IL-5-induced phosphorylation of Akt (0.30 ± 0.05 vs. 1.02 ± 0.21, *p* < 0.0001) and Erk1/2 (0.09 ± 0.02 vs. 1.16 ± 0.20, *p* < 0.0001) was significantly reduced (**Figure 4G**), indicating that Gab1 is required for IL-5-induced signal pathway activation.

**Figure 6:**
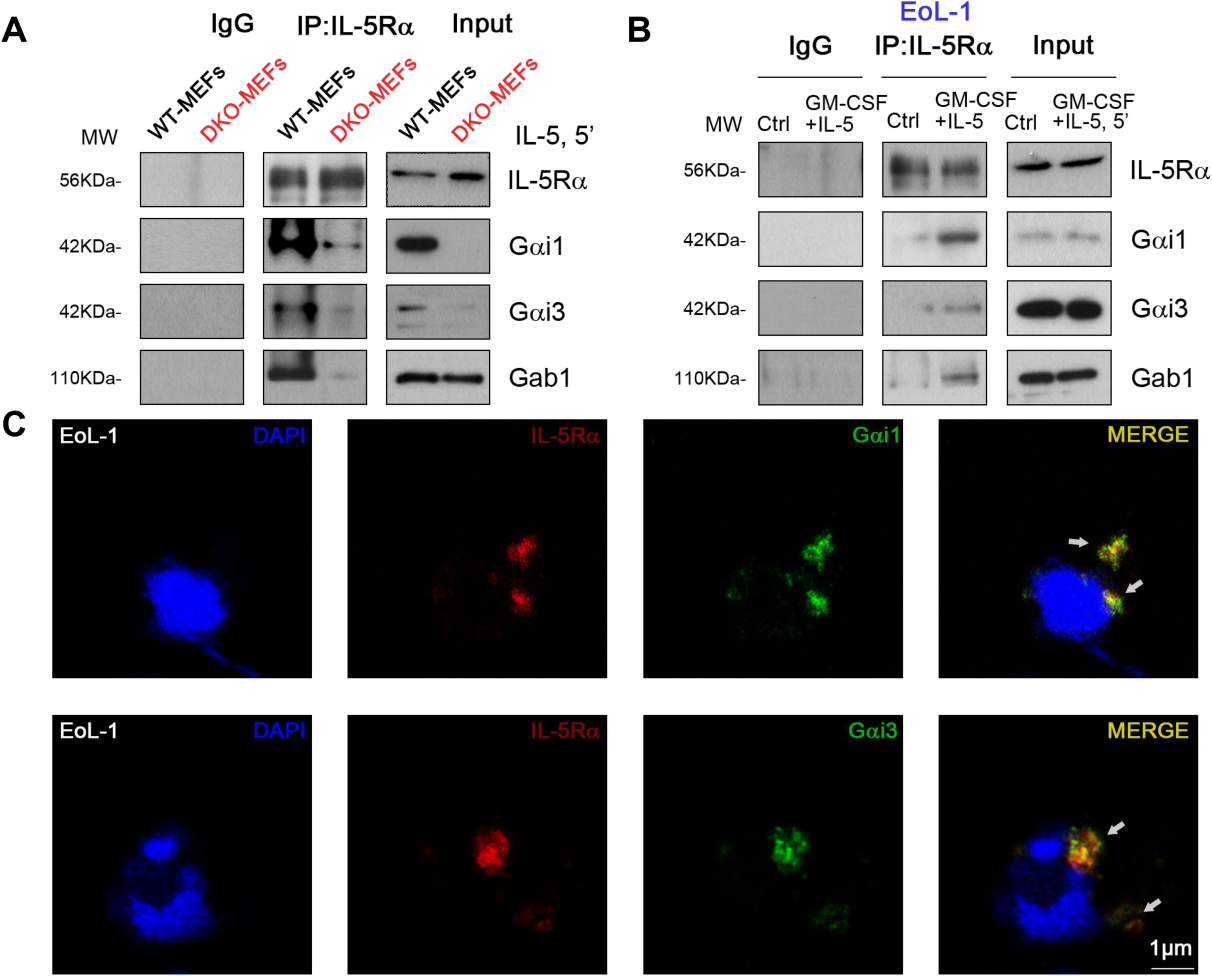
IL-5 induced Gαi1/3 association with activated IL-5 receptor and Gab1. WT and Gαi1/3 DKO MEFs were treated with IL-5 (25 ng/mL) for 5 min, the association between IL-5Rα, Gab1, and Gαi1/3 was tested by co-ip assay **(A)**. EoL-1 cells were treated with GM-CSF (50 ng/mL) and IL-5 (50 ng/mL) for 5 min, the association between IL-5Rα, Gab1, and Gαi1/3 was tested by co-ip **(B)**, and processed for confocal microscopy to detect the presence of Gαi1, Gαi3, and IL-5Rα **(C)**. Bar = 1 μm.

IL-5 activates STAT5, Akt, and Erk1/2 by coupling with IL-5R. Thus, we explored whether there is mutual binding between IL-5R, Gαi1, Gαi3, and Gab1. Co-IP results showed that the IL-5-induced association between IL-5Rα and Gab1 was largely inhibited in Gαi1/3 DKO MEFs (**Figure 6A**). To investigate the role of Gαi1/3 in IL-5-induced signaling in EoL-1 cells, we then stimulated the cells with IL-5. We found that IL-5Rα immunoprecipitated with Gαi1, Gαi3, and Gab1 (**Figure 6B**). Using confocal microscopy, we further observed colocalization between IL-5Rα and Gαi1/3 within 5 min of IL-5 treatment (**Figure 6C**).

### Inflammatory cell infiltration is largely impaired in Gαi1/3 DKO mice

**Figure 7:**
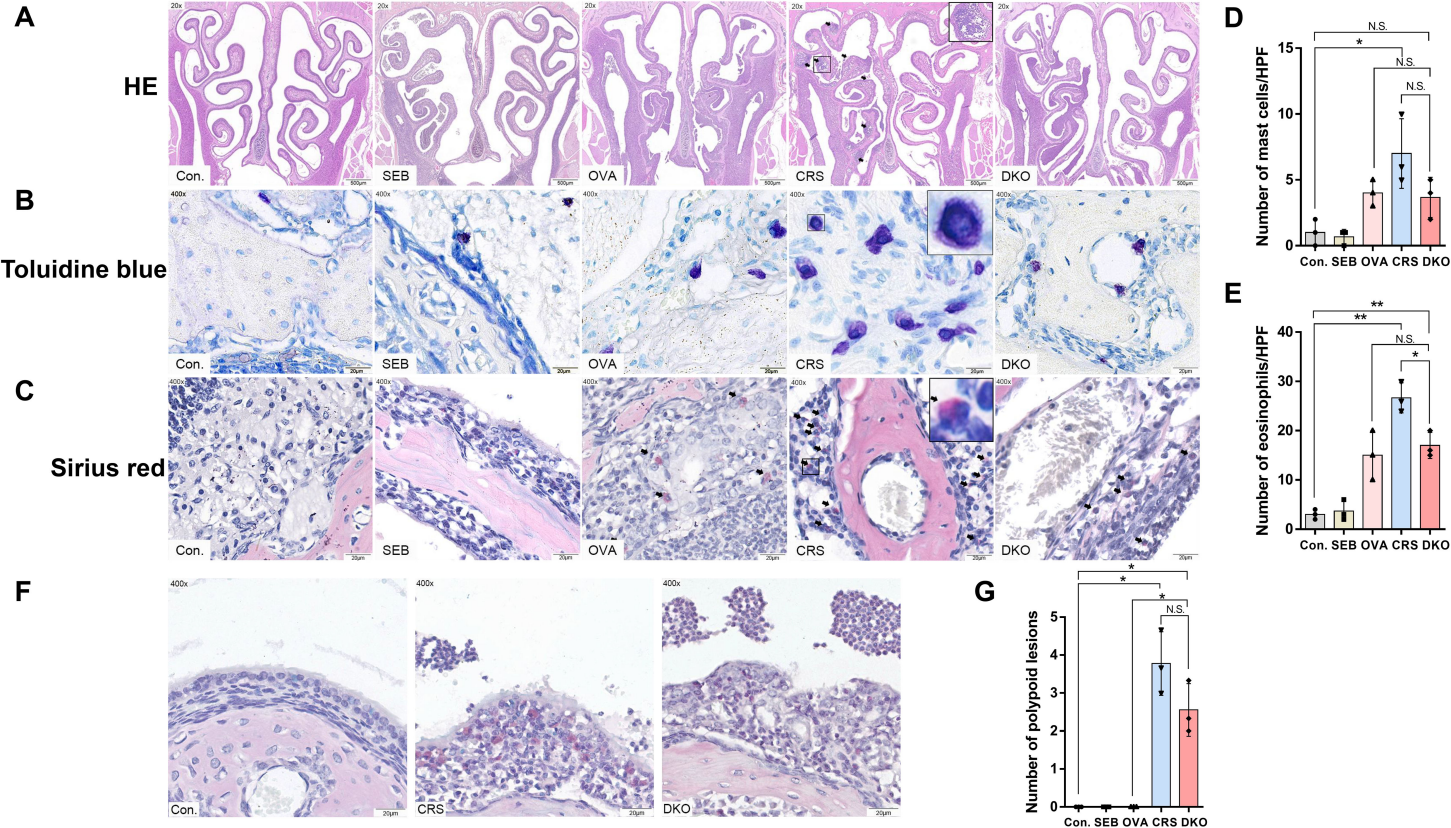
Impaired polypoid lesions and allergic inflammatory in Gαi1/3 DKO mice. Representative photographs of HE **(A)**, toluidine blue **(B)**, and sirius red staining **(C)** in control, SEB, OVA, CRS, and DKO groups. Nasal mucosal bulges (arrowhead) were detected in the mice treated with 12 weeks of OVA exposure plus 10 ng of SEB **(A)**. MCs (**(B)**, inset) and eosinophils (**(C)**, arrowhead) differentials according to groups. Comparison of MCs and eosinophil counts among the groups **(D, E)**. Epithelial disruption, inflammatory cell infiltration, and inflammatory exudation in different groups **(F)**. Comparison of polyp-like lesion counts among the groups **(G)**. MCs, mast cells; HPF, High-powered field; NS, no significant difference. *P <0.05 and **P <0.01.

The immune mechanism of eCRS is associated with the infiltration of inflammatory cells. Clinically, eCRS is more prone to polypoid changes. Based on previous studies, we used SEB and OVA to establish a mouse model of eCRSwNP (30). We then compared the effects of OVA and SEB sensitization and challenge on the development of eCRSwNP in WT (CRS group) and Gαi1/3 DKO mice (DKO group). In the OVA group (instillation of OVA only in WT mice), after 12 weeks of nasal OVA exposure, there was some exudate in the sinonasal cavities, subepithelial thickening was noted (**Figure 7A**), and scattered MCs and eosinophils were observed in the lamina propria (**Figures 7B, C**). In the CRS group, nasal polypoid lesions were detected in several areas after 12 weeks of nasal exposure to OVA with additional SEB. Exudates with crystal formations and surrounding eosinophils were observed in the sinus cavities (**Figures 7A, F**). In addition, more severe infiltration of MCs (7.00 ± 2.65 vs.1.00 ± 1.00, *p* = 0.0453, **Figure 7D**) and eosinophils (26.67 ± 3.06 vs. 3.00 ± 1.00, *p* = 0.0028, **Figure 7E**) were noted in the CRS group than the control group. The number of MCs (3.67 ± 1.53 vs. 7.00 ± 2.65, *p* = 0.1494, **Figure 7D**) and eosinophils (17.00 ± 2.65 vs. 26.67 ± 3.06, *p* = 0.0150, **Figure 7E**) were relatively low in the DKO group, compared to the CRS group, whereas there was no significant difference in the number of MCs. There was no significant difference in the number of nasal polypoid lesions between the DKO and CRS groups (3.78 ± 0.84 vs. 2.56 ± 0.69, *p* = 0.1262, **Figure 7G**).

### Defective type 2 inflammation in Gαi1/3 DKO mice

**Figure 8:**
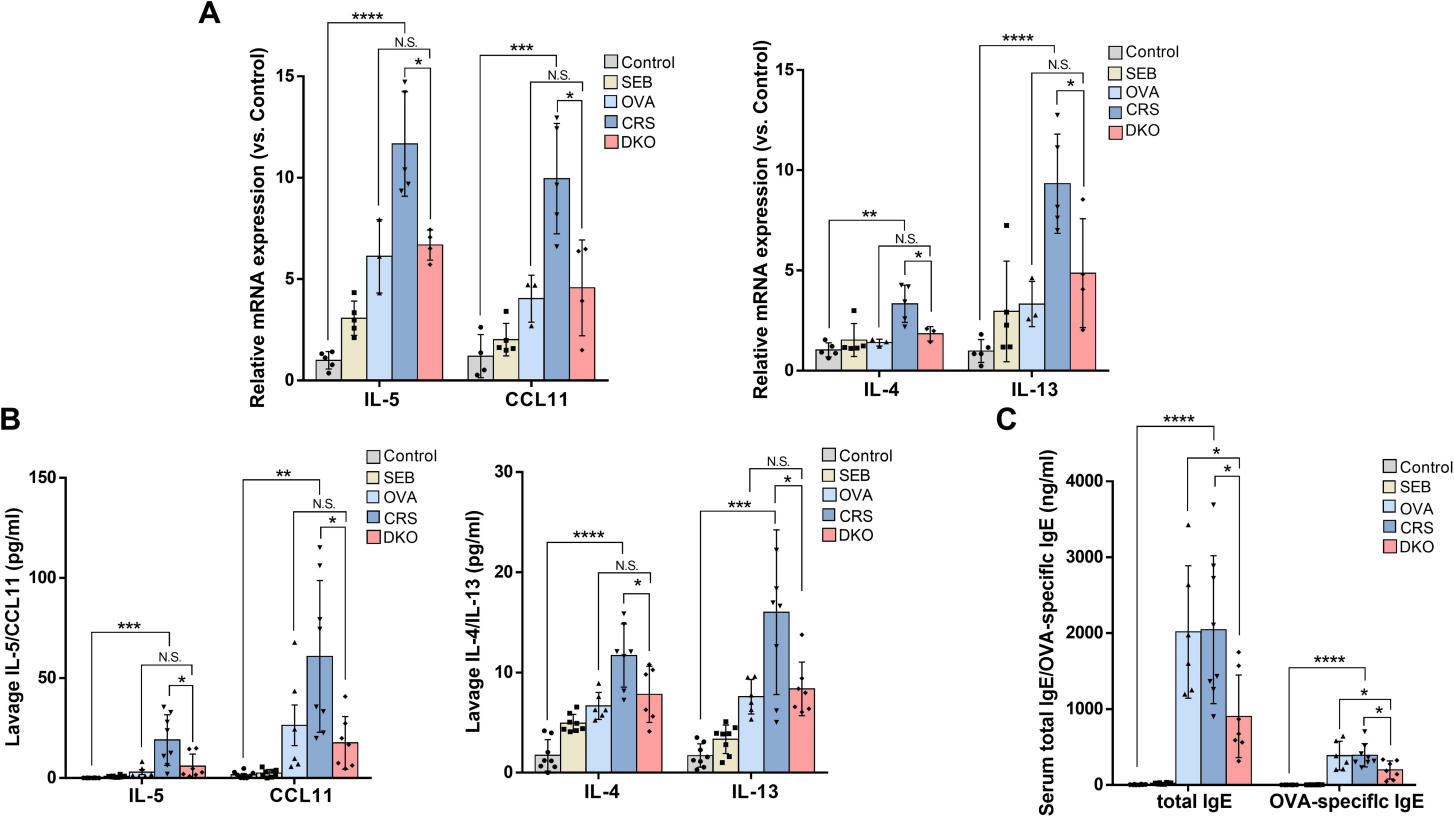
Defective type 2 immunity in Gαi1/3 DKO mice. mRNA levels of IL-4, IL-5, IL-13, and eotaxin in the nasal mucosa of mice were tested **(A)**. The levels of IL-4, IL-5, IL-13, and eotaxin from nasal lavage fluid **(B)**, and total IgE and OVA-specific IgE from serum **(C)** were measured by enzyme linked immunosorbent assay. *P <0.05, **P <0.01, ***P <0.001, and ****P <0.0001.

eCRS is pathophysiologically driven by type 2 inflammation and eosinophilic infiltration (5), a higher tissue eosinophil count, and increased eotaxin expression levels, suggesting greater eosinophil stimulation and chemotaxis with a higher degree of overall inflammation (6). As expected, OVA and SEB sensitization and challenge induced a striking increase in mRNA levels of type 2 cytokines (*IL-4*: 3.34 ± 0.93 vs. 1.04 ± 0.35, *p* = 0.0033; *IL-5*: 11.67 ± 2.58 vs. 1.00 ± 0.43, *p* < 0.0001; *IL-13*: 9.33 ± 2.48 vs. 0.98 ± 0.57, *p* < 0.0001) and eotaxin (*CCL11*: 9.96 ± 2.72 vs. 1.20 ± 1.06, *p* = 0.0009) in the nasal mucosa of WT mice (**Figure 8A**). Although increases in *IL-4*, *IL-5*, *IL-13*, and *CCL11* mRNA levels were also detected in the DKO group, they were obviously lower than those in group CRS (*IL-4*: 1.84 ± 0.36 vs. 3.34 ± 0.93, p = 0.0203; *IL-5*: 6.68 ± 0.75 vs. 11.67 ± 2.58, *p* = 0.0100; *IL-13*: 4.87 ± 2.72 vs. 9.33 ± 2.48, *p* = 0.0423; *CCL11*: 4.57 ± 2.36 vs. 9.96 ± 2.72, *p* = 0.0159, **Figure 8A**).

We then examined the levels of IL-4, IL-5, IL-13, and eotaxin in the nasal lavage fluid. In nasal lavage fluid, the levels of type 2 cytokines (IL-4: 7.82 ± 2.82 vs. 11.69 ± 3.15, *p* = 0.0394; IL-5: 5.88 ± 6.12 vs. 18.97 ± 12.67, *p* = 0.0259; IL-13: 8.36 ± 2.68 vs. 16.01 ± 8.22, *p* = 0.0356) and eotaxin (17.56 ± 13.14 vs. 60.75 ± 37.91, *p* = 0.0305) were lower in the DKO group than the CRS group (**Figure 8B**). These data further indicated inhibition of the Th2 response in OVA + SEB–treated Gαi1/3 DKO mice. In addition, we found that the CRS group produced a large amount of serum IgE (2,045.57 ± 974.12 vs. 4.39 ± 4.56, *p* < 0.0001) and OVA-specific IgE (391.85 ± 152.48 vs. 0.01 ± 0.02, *p* < 0.0001, **Figure 8C**) compare to control group. Serum IgE (903.68 ± 544.34 vs. 2,045.57 ± 974.12, *p* = 0.0156) and OVA-specific IgE (196.65 ± 119.67 vs. 391.85 ± 152.48, *p* = 0.0159) production was, however, attenuated in the DKO group than the CRS group (**Figure 8C**).

## Discussion

To the best of our knowledge, this is the first study to show that Gαi1 and Gαi3 are upregulated in the nasal tissue of CRS patients, especially in eCRS patients. We found that high nasal tissue Gαi1/3 levels were linked to high disease severity and allergic conditions, as well as high levels of eosinophil infiltration in CRS patients. Deficiency of Gαi1 and Gαi3 in EoL-1 cells results in resistance to IL-5 + GM-CSF-induced degranulation and proliferation via impaired activation of signaling pathways. Gαi1 and Gαi3 were required for IL-5 and GM-CSF–induced IL-5Rα endocytosis and Gab1 recruitment in EoL-1 cells. Then, using an eCRSwNP murine model, we found that OVA + SEB–induced nasal eosinophilia infiltration and type 2 cytokine release were largely impaired in Gαi1 and Gαi3 DKO mice, compared to WT mice. Our findings indicated that Gαi1 and Gαi3 are novel and key mediators of eCRS pathogenesis.

To the best of our knowledge, the functional relevance of Gαi1/3 in human CRS diseases has rarely been studied. Endoscopically, diffuse polyp growth and thick eosinophilic mucin are observed in eCRSwNP patients (5, 31, 32). Based on our observations, there was no significant difference in the MOD of Gαi1/3 between CRSsNP and eCRSwNP. Protein levels of Gαi1/3 were notably higher in eCRSwNP patients compared to non-eCRSwNP patients suggesting that they may play a more important role in eosinophilic lesions rather than being the cause or result of polypogenesis. Of note, the prevalence of allergies in CRS patients may vary by phenotype; however, eCRS is more strongly associated than non-eCRS with type 2 inflammation and allergic inflammation (33, 34).

Previous research has indicated that that Gαi1/3 is expressed in immune cells such as MCs, macrophages, and basophils, all of which play a role in the progression of CRS disorders (10–13). In our continuous sections, we observed a discrepancy between the regions of eosinophils and Gαi1/3 expression based on HE staining and immunohistochemical staining for Gαi1/3 (**Figure 1A**). Therefore, we speculate that Gαi1/3 may also be expressed on the surface of other infiltrating immune cells, which requires further investigation. Immunohistochemical staining analysis revealed the presence of Gαi1/3 expression in both immune cells and epithelial mucosa, with a particularly pronounced expression observed in the mucosa of patients with eCRS. This may be attributed to the migration of inflammatory cells towards the site of mucosal inflammation. Previous studies have also confirmed the role of Gαi1/3 expression in fibroblasts and skin keratinocytes in the healing process of skin tissue (21). Further research is warranted to determine the effects of these two proteins on inflammation and damage to the nasal mucosa.

Gαi1/3 genomic analysis via bioinformatics and gene sequencing showed no significant differences between control and CRS groups, or between eCRSwNP and non-eCRSwNP groups (**Supplementary Figure S4**). We observed that Gαi1/3 proteins were highly expressed in eCRS than non-eCRS. One possible explanation for this is that post-transcriptional modifications or protein degradation may play a role in the observed differential protein expression levels. Another possibility is that changes in the translation efficiency of mRNA or the regulation of protein synthesis or degradation contributed to the differences in protein levels between the two groups. The regulation of transcription, transcript degradation, translation, and protein degradation contributes significantly to variations in protein concentration (35). Our findings highlight the need to explore the role of post-transcriptional modification and protein regulation in modulating cellular responses. Future research should employ a hybrid approach, incorporating transcriptomics, proteomics, and functional studies to gain a more comprehensive understanding of Gαi1/3 expression levels in response to different stimuli in CRS.

We then found high local eosinophil infiltration and blood eosinophil count were associated with increased nasal tissue Gαi1/3 levels in CRS, especially in eCRS. Although previous findings suggest a regulatory role of Gαi1/3 on allergy-associated immune cells and type 2 inflammation (10, 11), its effect on Th2 cells and eosinophils remains unknown. We found that the nasal tissue Gαi1/3 levels are higher in CRS patients with atopy than in those without atopy. Allergic complications, such as allergic rhinitis and asthma, are more frequently found in CRS patients with eosinophilia than in those without eosinophilia (36). Biologic agents that suppress type 2 inflammation may, suppress the inflammation, reverse the remodeling and limit recurrence, thereby altering the clinical course of the most severe CRS phenotypes (4). Thus, Gαi1 and Gαi3 are potential targets for treating CRS, especially eCRS. Lund-Mackay CT scores are associated with CRS symptom severity (37, 38). Correlation analyses showed a positive association between Lund-Mackay CT scores and nasal tissue Gαi1/3 levels, suggesting their potential as indicators of CRS severity.

Additionally, previous research indicate that Gαi1 and Gαi3 participate in regulating the IgE-FcεRI-mediated degranulation of MCs (11). In this study, we did not investigate the functional effects of Gαi1/3 and IL-5 on MCs. In future research, we plan to include immune cells related to CRS inflammation, such as MCs and macrophages, to examine the regulatory effects of IL-5 and Gαi1/3 on these cells and how they influence disease progression. These findings prompted us to further explore the contribution of Gαi1/3 to targeting eosinophils and type 2 inflammation in the pathogenesis of eCRS.

CRSsNP is characterized by predominantly neutrophilic inflammation with increased levels of type 1 cytokines (39). CRSsNPs can also be classified into type 2 and non-type 2 inflammatory endotypes based on their pathogenesis. Therefore, comprehensive immunological and pathological data collection is necessary to classify the diseases and explore the role of Gαi1/3 in different endotypes of CRSsNP. The expression of Gαi1/3 in patients with CRS may be influenced by factors such as tobacco smoke, allergies, infections, genetic predisposition, environmental pollution, living conditions, underlying diseases, and immune system abnormalities, though the specific role remains unclear, requiring further clinical data collection and analysis.

Activated CD4+ T cells, particularly the Th2 cell subset, are one of the major producers of IL-5. IL-5 plays a key role in differentiation, development, and survival of eosinophils (28). Nasal administration of recombinant IL-5 induces selective recruitment of eosinophils to the mucosa. At the mechanistic level, IL-5 signaling in mature eosinophils activates several signaling molecules, such as the STAT5 and ERK pathways (40). However, to date, studies on eosinophils have been prevented by the small number of cells that can be obtained from the peripheral blood of healthy people and the inability to expand eosinophils *in vitro* (41). MEFs are multifunctional cells that participate in various signaling pathways involved in many cellular processes. And EoL-1 cells have been used as a model cell line to study eosinophilic functions (42, 43). Moreover, EoL-1 cells express IL-5Rα, a critically important receptor for the survival and activation of eosinophils, which is likewise expressed in human eosinophils (44). Therefore, we preliminarily investigated the role of Gαi1/3 in IL-5 mediated signaling in MEFs and EoL-1 cells. We found that Gαi1 and Gαi3 were required for IL-5-induced Akt and Erk activation in MEFs. However, we did not analyze the STAT5 pathway or total STAT5 protein in MEFs by western blotting. We speculated that the STAT5 levels in MEFs were low and difficult to detect. Therefore, we validated this pathway in EoL-1 cells. Prior research has shown that Gαi2 SKO failed to significantly affect IL-4-induced Akt-S6K1 and Erk1/2 phosphorylation in MEFs (10). Furthermore, related studies indicated that Gαi1 and Gαi3 were abundantly expressed in the immune system and were involved in the signaling processes of various receptors, unlike Gαi2 (8–11). Notably, we stimulated Gαi2 knockout MEFs with IL-5 and found that the activation of downstream signaling pathways remained unaffected (**Figure 4D**). Therefore, we did not conduct further research on Gαi2 in subsequent experiments. We observed that Gαi1/3 silencing in EoL-1 cells blocked STAT5 phosphorylation, significantly impairing degranulation, differentiation, and proliferation of the cells. JAK2 is associated with IL-5R (45), but we did not further explore the relationship between JAK2 and Gαi1/3 in the present study.

Previous studies have indicated that Gab1 is an adaptor protein for Gαi proteins, which can form complexes with Gαi1/3 to mediate downstream signaling (21, 22). IL-5 signals via the IL-5R α chain and β chain complex. Eosinophils highly express IL-5Rα on their surface (46). The present study reveals a unique mechanism for the role of Gαi proteins in IL-5R signaling. Our results showed that Gab1, Gαi1, and Gαi3 physically associated with the intracellular domain of IL-5Rα, and this was essential for IL-5Rα endocytosis and STAT5, Erk, and Akt activation in MEFs and EoL-1 cells. This study lacks direct evidence to prove the effect of Gαi1/3 on the endocytic process of IL-5Rα, which is a limitation of our research. Future work should include additional experiments for further validation, such as endocytosis kinetics studies utilizing fluorescently labeled IL-5Rα antibodies or ligands. By conducting time-lapse experiments, we can observe the presence or absence of Gαi1/3 and analyze the endocytosis rate and degree of internalization of IL-5Rα using flow cytometry or confocal microscopy. Additionally, endocytosis inhibition experiments should be performed by treating cells with specific inhibitors to examine changes in IL-5Rα endocytosis in the presence or absence of Gαi1/3. By comparing the differences between various treatment groups, we can infer the specific role of Gαi1/3 in the endocytic process. In this study, we confirmed the co-localization of Gαi1/3 and IL-5Rα using immunofluorescence double staining. Additional experiments, such as flow cytometry, would further validate these findings, which is a limitation of our current research. We will address this in our future studies. IL-5-induced signaling requires the entry of activated IL-5R into the intracellular compartment, as Co-IP of key signaling molecules with IL-5R is completely blocked when either of the endocytic pathways is inhibited (47). IL-5 has been considered a therapeutic target for allergic diseases because of the exclusive IL-5R expression in eosinophils and human basophils and its critical role in eosinophilopoiesis (48). In this study, due to the challenges of extracting human eosinophils, we only utilized two model cell lines, EoL-1 and MEFs, for cellular mechanism experiments. The interaction between Gαi1/3 and IL-5Rα in signaling could also be investigated using mouse bone marrow-derived eosinophils, highlighting a limitation in our research. In future studies, we will conduct differentiation experiments of bone marrow-derived eosinophils (BMDE) using DKO and WT mice, and analyze the populations of eosinophils and eosinophil progenitors in the bone marrow of CRS models through flow cytometry, to further elucidate the relationship between Gαi1/3 and IL-5Rα in eosinophilia.

We believe that the functional compensation observed between Gαi1 and Gαi3 results from multiple contributing factors. Structural Similarity: Gαi1 and Gαi3 are members of the G protein α subunit family and exhibit a high degree of structural similarity. This structural similarity may result in a certain level of redundancy in their functions. Overlapping Signaling Pathways: Both Gαi1 and Gαi3 are involved in regulating several signaling pathways, including but not limited to the IL-5 signaling pathway. Within these pathways, they may play similar or complementary roles. Cell Type Specificity: The expression levels and functions of Gαi1 and Gαi3 may differ across various cell types. However, in specific cell types, such as eosinophils, they may jointly participate in the same physiological or pathological processes. In such contexts, if one subunit is inhibited or deleted, the other subunit can partially compensate for its function, maintaining normal physiological function of the cell. This finding not only enhances our understanding of the roles of Gαi1 and Gαi3 in the pathophysiology of CRS but also offers potential new targets for future therapeutic interventions.

Th2 cytokines recruit leukocytes to sites of inflammation and are essential for IgE synthesis and eosinophilia (49). Type 2 inflammation is characterized by the activation and recruitment of eosinophils and MCs (4). MCs in eCRS produce IL-5 after stimulation with TSLP and IL-1β, promoting eosinophil activation and proliferation (50). The infiltration, activation, and mediator release of eosinophils, MCs, and basophils are important for polyp formation (19). We observed type 2 inflammatory factors, polyp formation, and inflammatory cell infiltration in WT mice. These data agree well with those of a long-term study by Kim et al. (51). Clinical data suggest that Tryptase+ cells are significantly increased in the eCRSwNP group compared to the other two groups. However, in the animal model shown in **Figure 7**, MCs in the CRS group do not exhibit significant changes. OVA combined with SEB induces eCRS in mice, exhibiting Th2 type immunoreactivity and an eosinophilic inflammatory response similar to human eCRS. However, this model has limitations (24, 51); it primarily involves antigens and bacterial toxins, which do not encompass all eCRS induction factors. The OVA induced polypoid transformation in mouse models is characterized by eosinophil infiltration (24), but MCs are crucial in human nasal polyps. Thus, transferring findings from animal studies to human diseases requires careful consideration, highlighting ongoing challenges in identifying the optimal animal model for eCRS. In the current study, using the eCRSwNP murine model (24), we found that the levels of eotaxin and type 2 cytokines, including IL-4, IL-5, and IL-13, in local tissues and nasal lavage fluid were lower in Gαi1 and Gαi3 DKO mice, compare those in the CRS group. The degree of eosinophilia was diminished in DKO mice. Our data suggest that Gαi1 and Gαi3 are novel and key mediators of allergic and type 2 inflammation pathogenesis. Targeting Gαi1/3 may provide a new therapeutic modality for CRS, particularly eCRS.

SEB and its enterotoxins are involved in stimulating the Th2 system to promote IgE production and eosinophil infiltration through various pathways (7). Braga et al. (52) have successfully induced eCRS in rabbits by using SEB alone. The role of Gαi1/3 protein in the induction and severity of SEB-induced sinus mucosal inflammation and disease is unclear. Studies have shown that anti-TNFα antibodies protect against SEB-induced tissue and organ damage (53). In our mouse study, we aimed to explore the mechanism of action of Gαi1/3 in the development of eCRS induced by both SEB and OVA, and did not focus on the impact of using SEB alone, therefore, we did not further subgroup the DKO mice, which is a limitation in our study. However, after data analysis, we realized the significance of this aspect in our research. It is necessary to further investigate the mechanism of SEB in disease progression by using DKO mice injection drugs such as OVA or SEB to explore whether Gαi1/3 protein acts in specific pathways, such as the TNF-α pathway. In this study, we employed Gαi1/3 DKO mice, instead of eosinophil-specific Gαi1/3-deficient mice, to evaluate the role of Gαi1/3 in a murine model of CRS. Besides, using eosinophil-specific Gαi1/3-deficient mice to evaluate the role of Gαi1/3 in CRS can further illustrate the role of Gαi1/3 in eosinophils of CRS patients or CRS mouse models. Specifically, CRISPR/Cas9 technology can be utilized to clone target sequences and introduce them into mouse embryonic cells, and screen and validate them to establish a *Gαi1*/*3*-gene-knockout mouse model specifically in eosinophilic granulocytes, to study the extent to which Gαi1/3 plays a role in CRS pathology through eosinophilic granulocytes.

### Conclusions

In summary, we observed that high levels of Gαi1/3 in local nasal tissues are closely associated with high levels of eosinophil, MC, and IgE^+^ cell infiltration, and atopy in CRS. Our data show that, activation of STAT5, Akt, and Erk1/2 in eosinophils, in part via Gαi1/3 binding to IL-5R, mediates the IL-5 signaling pathway. Using a eCRSwNP murine model, we found that type 2 inflammation, immune cell infiltration, and polyp-like lesions are largely impaired in Gαi1/3 DKO mice. Given the abovementioned findings, we conclude that blocking the activation of eosinophils by targeting Gαi1/3, especially via the manipulation of IL-5R pathways targeting eosinophil activation, may be useful for the treatment of CRS with high eosinophil counts and alleviate the type 2 inflammatory response.

## Data Availability

The raw data supporting the conclusions of this article will be made available by the authors, without undue reservation.

## Supplementary material

Supplementary Figure 1 Protocol for ovalbumin sensitization and administration of Staphylococcus aureus enterotoxin B.

Supplementary Figure 2 Increased numbers of eosinophils in patients with eCRSwNP. Nasal mucosa tissues were fixed and subjected to HE staining **(A)**. Bar = 20 μm. Insets show a higher magnification of the outlined area. Arrows denote eosinophils. Quantification of eosinophils in local nasal mucosal tissues **(B)**. **P <0.01 and ****P <0.0001.

Supplementary Figure 3 Increased numbers of mucosal tryptase^+^ cells and IgE^+^ cells in patients with eCRSwNP. Representative immunostaining photomicrographs show tryptase^+^ cells and IgE^+^ cells in nasal mucosa **(A)**. Bar = 100 μm. Quantification of tryptase^+^ cells and IgE^+^ cells in local nasal mucosal tissues **(B)**. Correlations between numbers of tryptase^+^ cells and IgE^+^ cells in patients **(C)**. Correlations of Lund-Mackay CT scores with numbers of tryptase^+^ cells and IgE^+^ cells in patients **(D, E)**. HPF, High-powered field; eCRSwNP, eosinophilic CRSwNP; non-eCRSwNP, non-eosinophilic CRSwNP. ****P <0.0001.

Supplementary Figure 4 Genomic analysis of Gαi1/3. The original expression levels of Gαi1 and Gαi3 in the control group and CRS patients’ nasal tissues were compared using GSE10406 and GSE36830 datasets **(A, B, D, E)**. Besides, Gαi1/3 tissue sequencing was performed on nasal polyps tissues collected from 6 eCRSwNP and 5 non-eCRSwNP patients **(C, F)**. There was no significant difference in gene expression between the two groups **(C, F)**. GNAI1, Gαi1 protein corresponding gene name. GNAI3, Gαi3 protein corresponding gene name. NS, no significant difference. eCRSwNP, eosinophilic CRSwNP; non-eCRSwNP, non-eosinophilic.

Supplementary Figure 5 The uncropped blotting images of the study.
