# Supplementary figures and images for: Gαi1/3 signaling mediates IL-5-induced eosinophil activation and type 2 inflammation in eosinophilic chronic rhinosinusitis
Source: Front Immunol. 2025 Jan 7;15:1460104. doi: 10.3389/fimmu.2024.1460104 (PMC11746084; doi:10.3389/fimmu.2024.1460104)

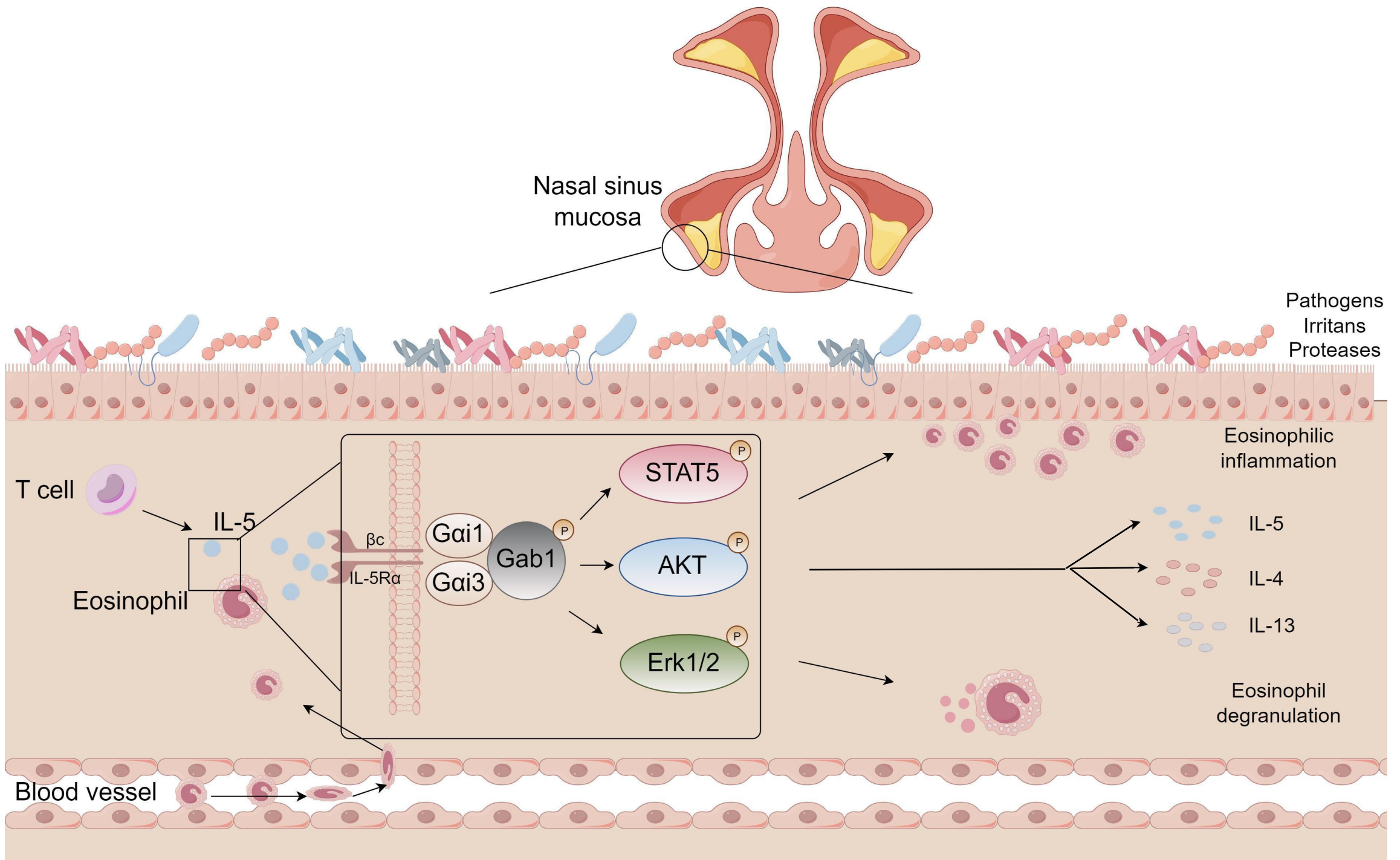

Supplement: Supplementary file 1 [file DataSheet1.pdf]
