# Supplementary material for: Gαi1/3 signaling mediates IL-5-induced eosinophil activation and type 2 inflammation in eosinophilic chronic rhinosinusitis
Source: Front Immunol. 2025 Jan 7;15:1460104. doi: 10.3389/fimmu.2024.1460104 (PMC11746084; doi:10.3389/fimmu.2024.1460104)
Supplement: Supplementary Figure 1 — Protocol for ovalbumin sensitization and administration of Staphylococcus aureus enterotoxin B. [file DataSheet2.pdf]

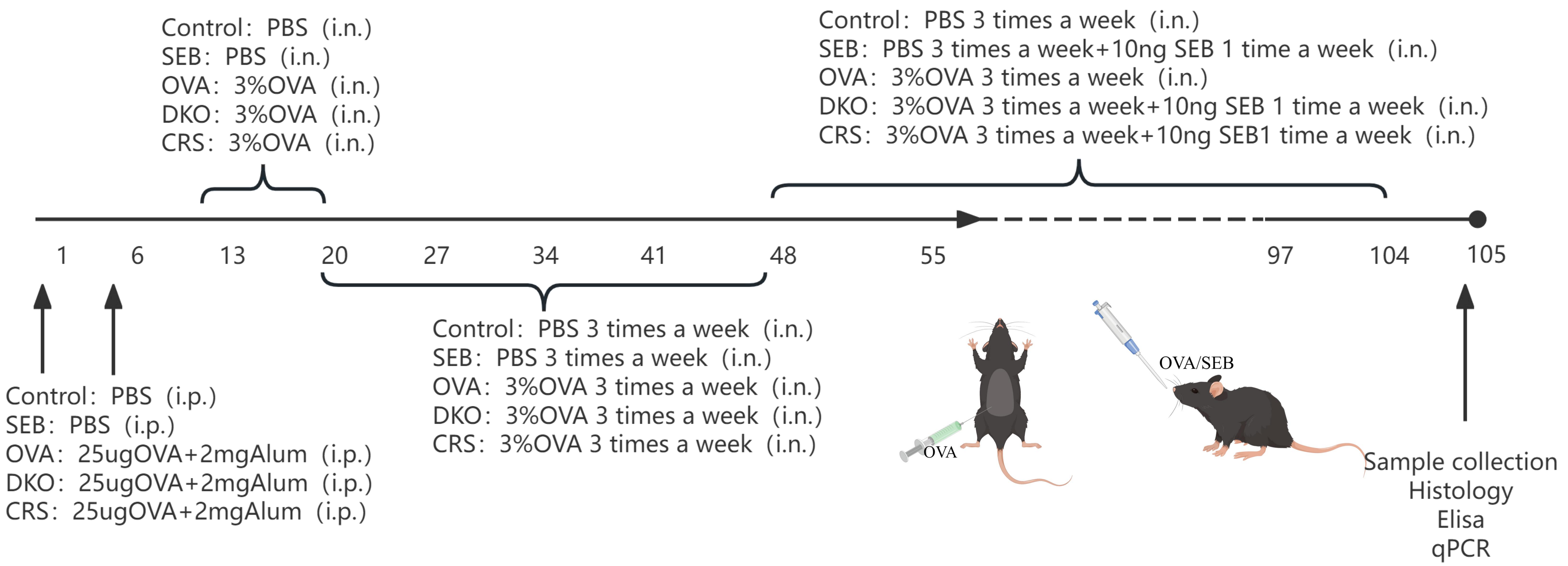

Supplementary figure 1

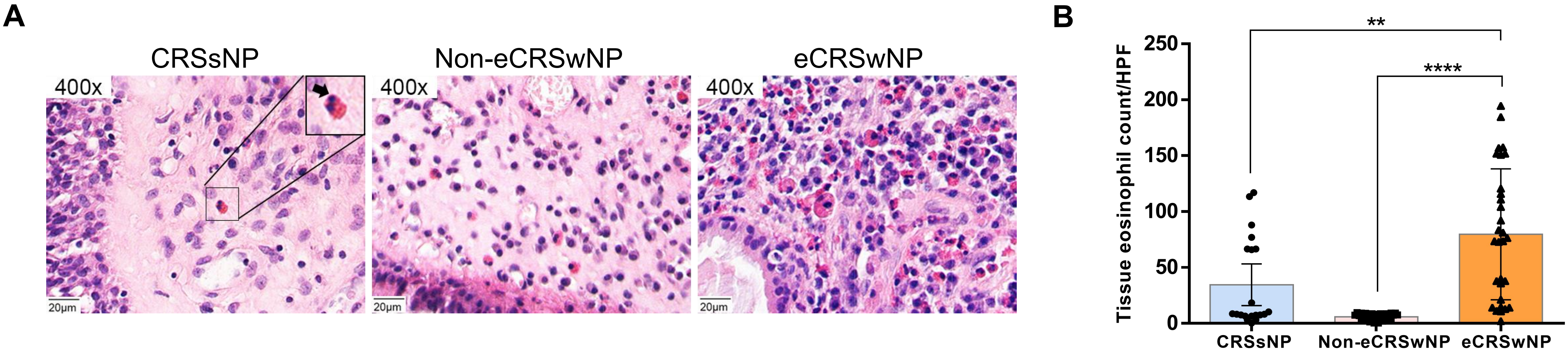

Supplementary figure 2

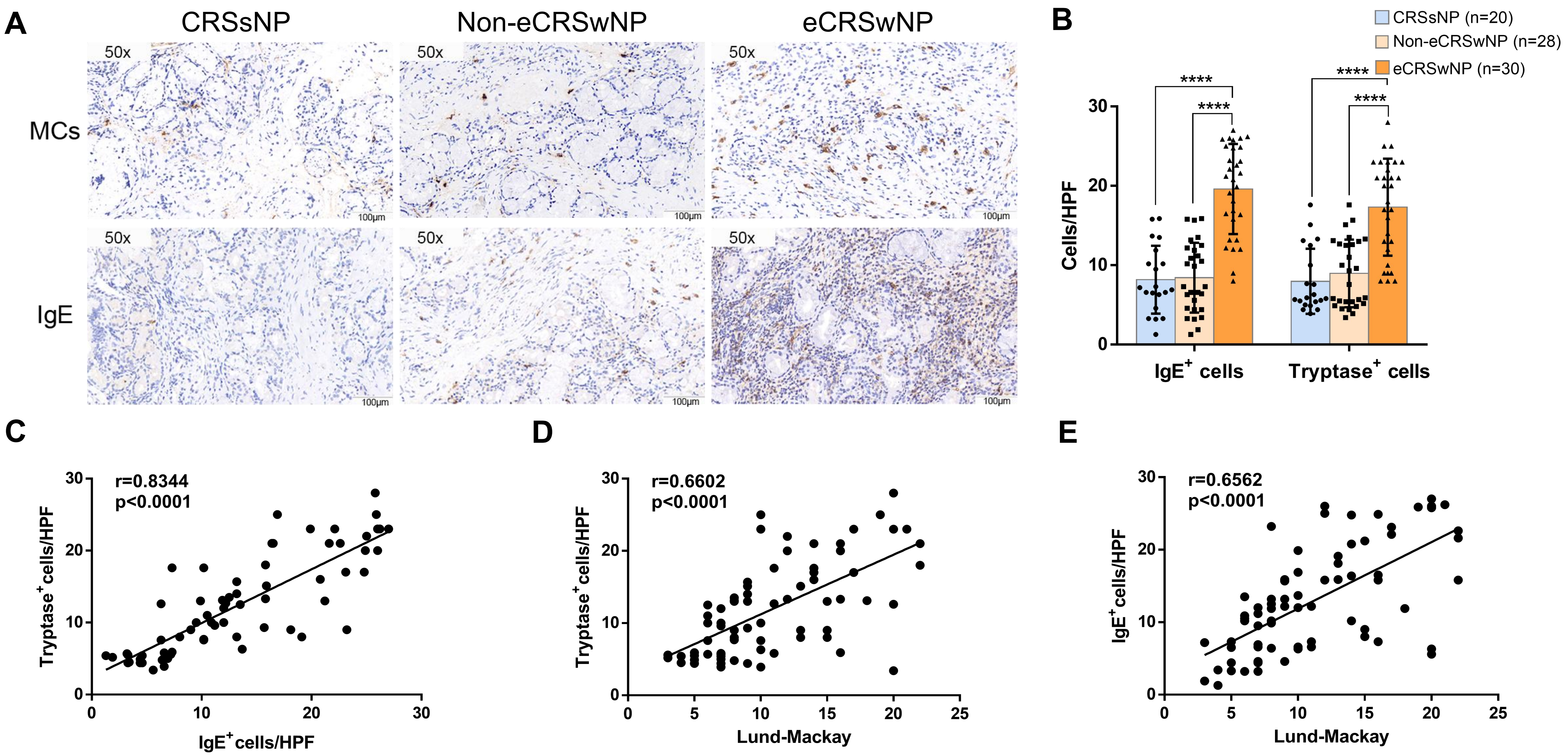

Supplementary figure 3

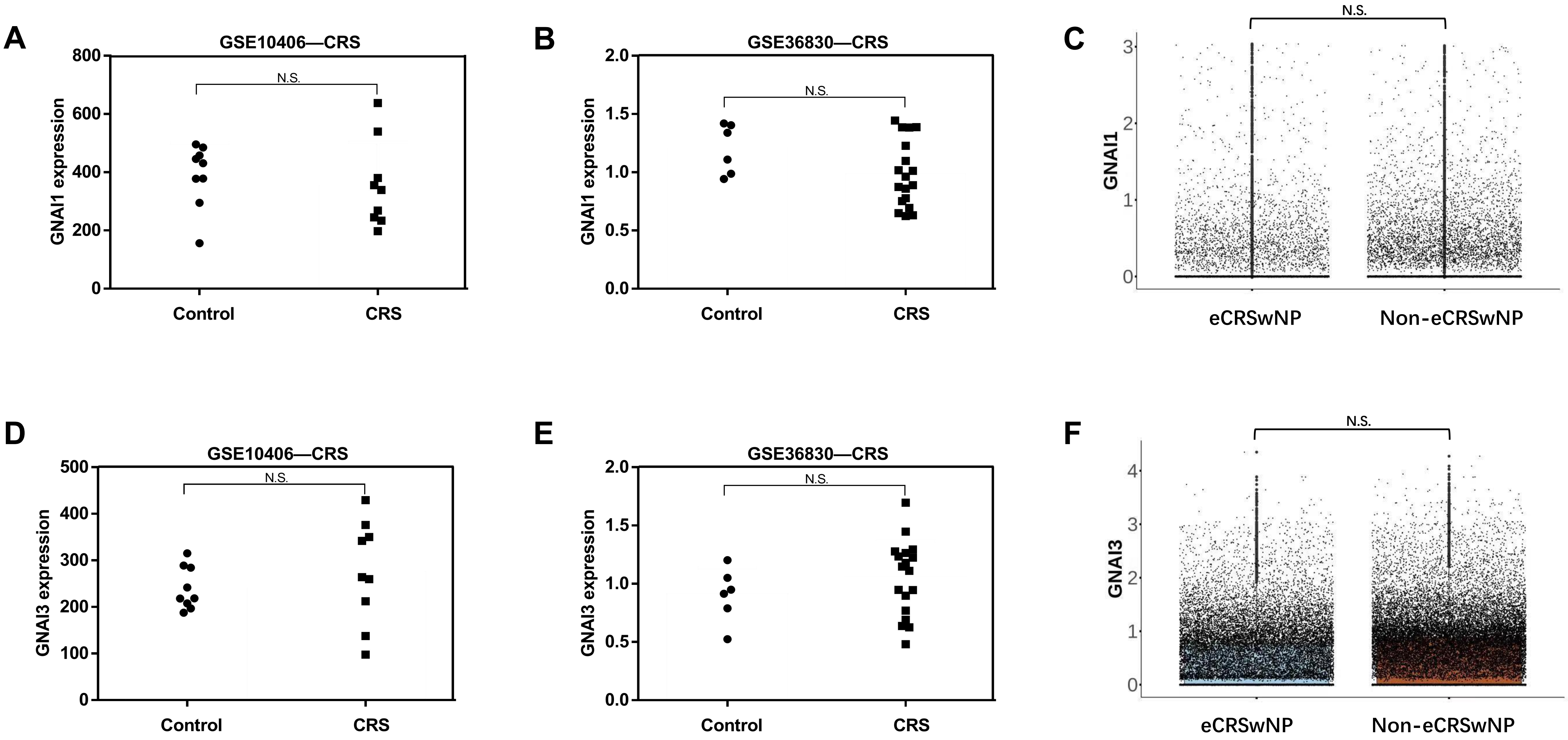

Supplementary figure 4

Figure 1

A

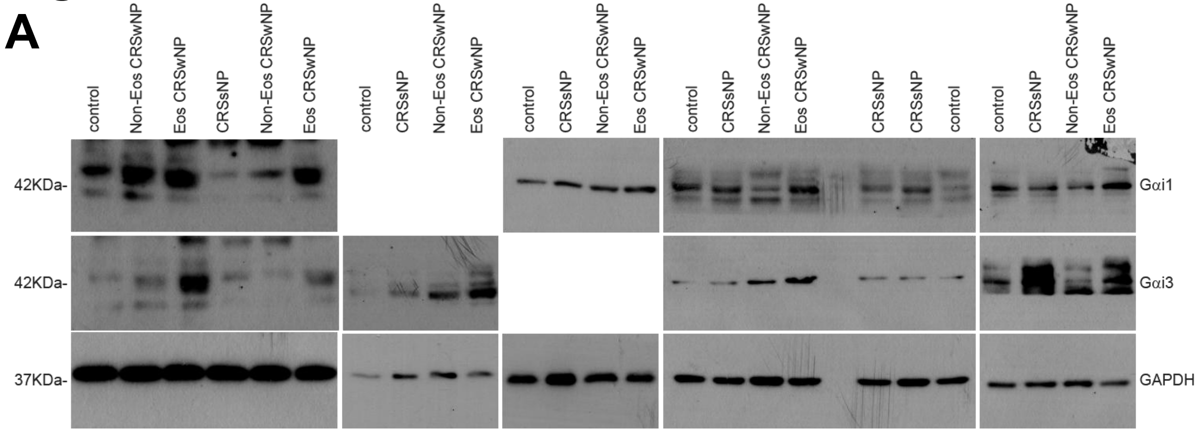

Figure 3

A

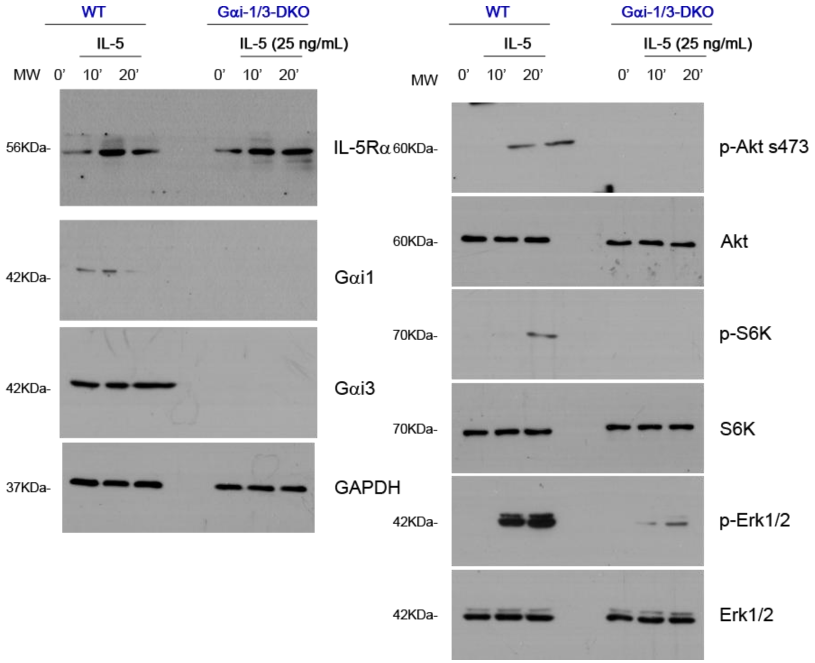

B

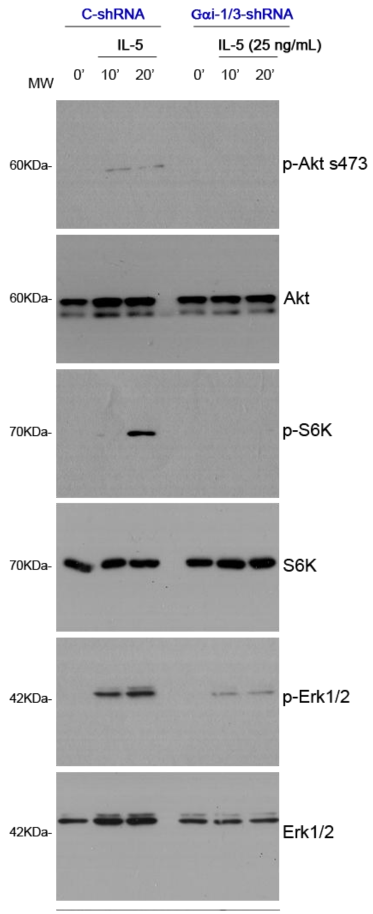

C

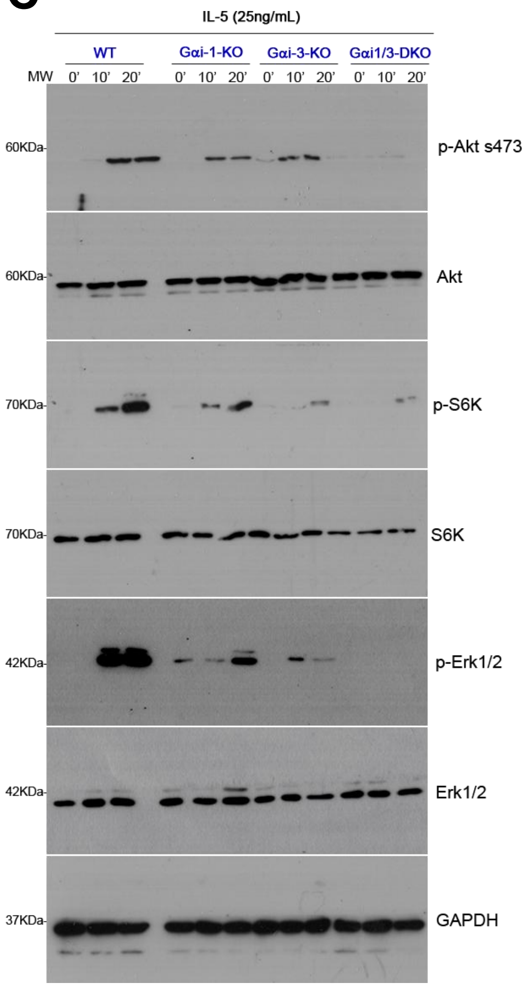

D

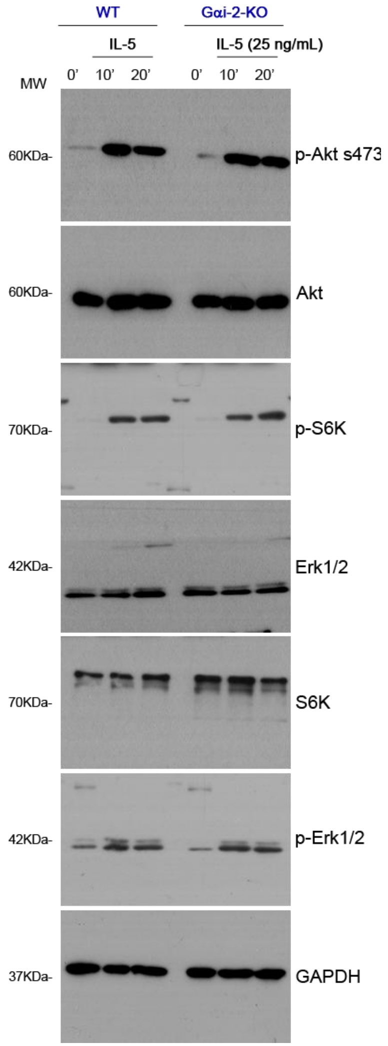

Supplementary figure 5

Figure 3

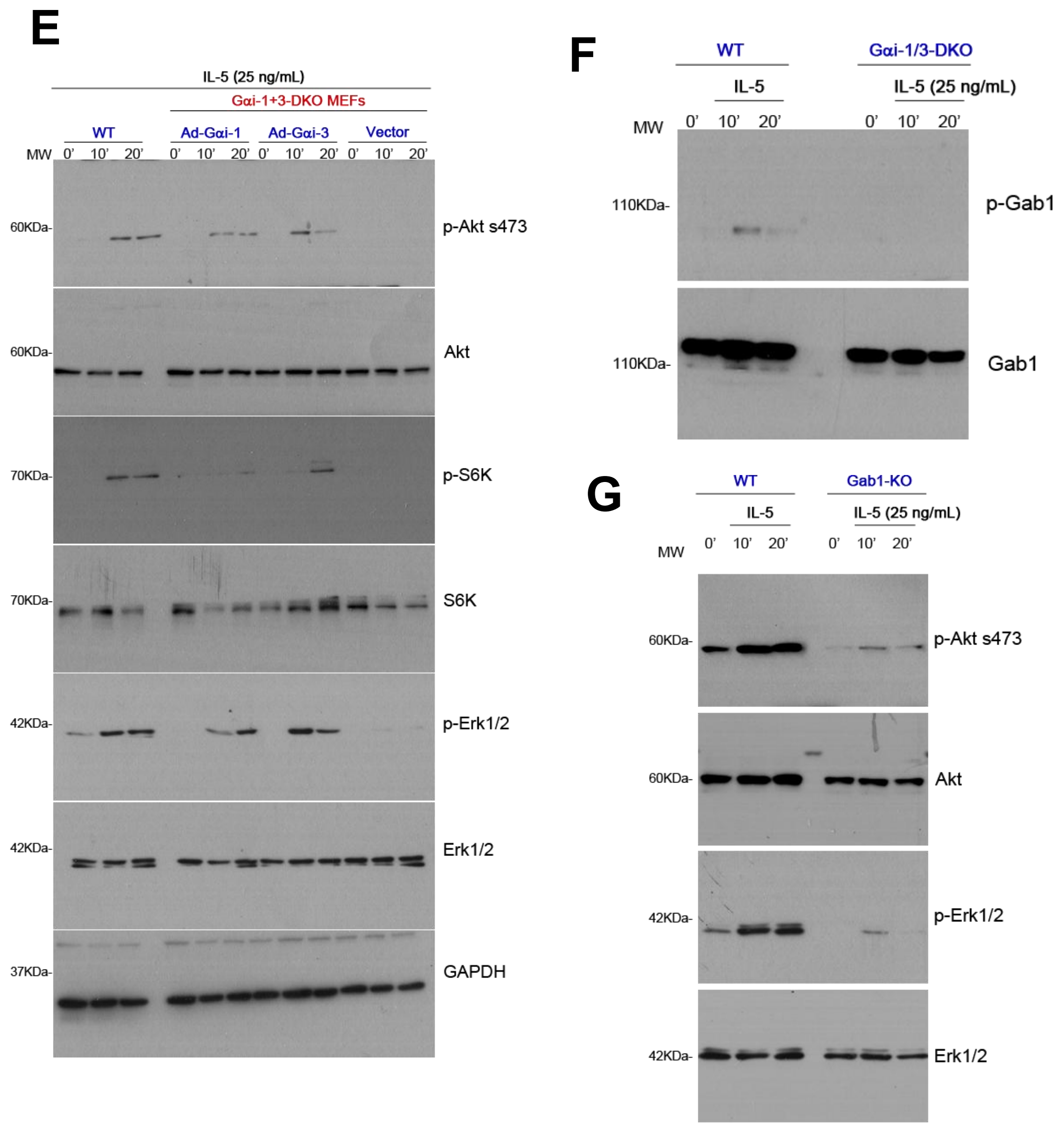

Figure 4

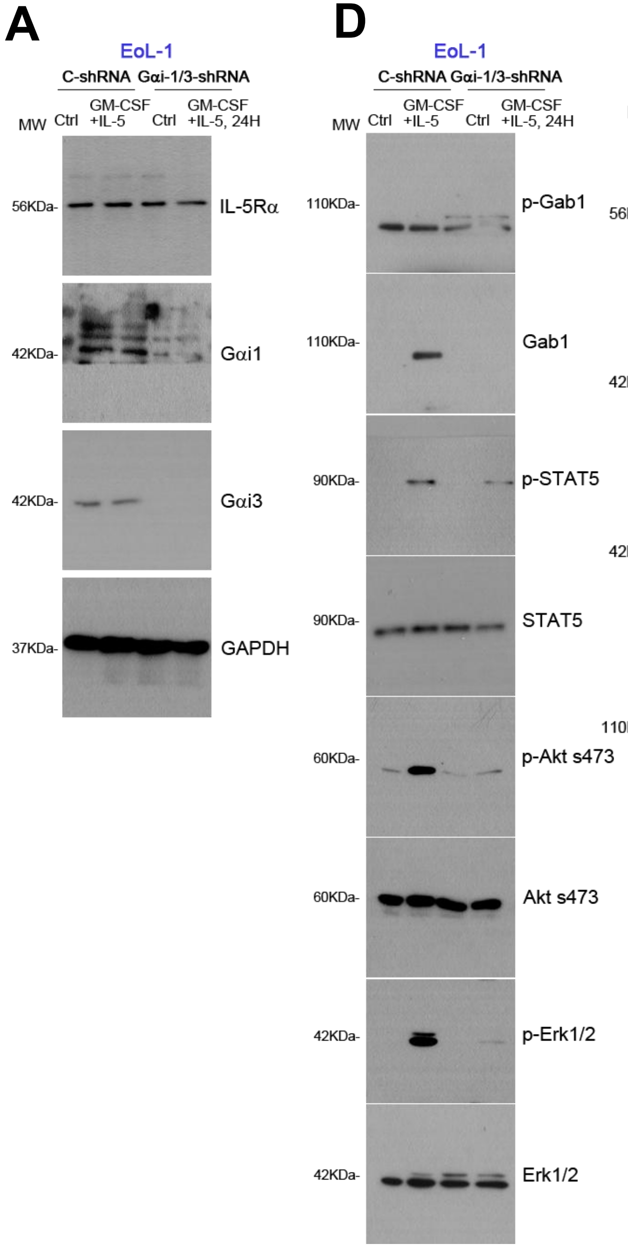

Figure 5

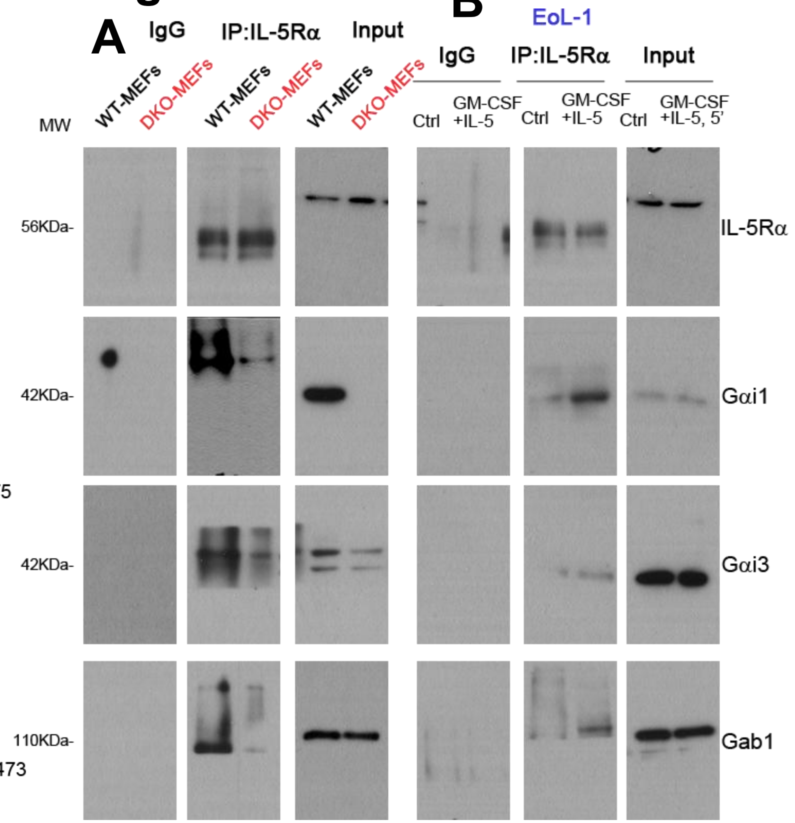

Supplementary figure 5
